# Radiotherapy-chemodynamic cancer therapy using bismuth-based nanoparticles: a synergistic approach for enhanced cancer treatment

**DOI:** 10.1039/d5ra03984c

**Published:** 2025-09-11

**Authors:** Charles Ifeanyi Aghanwa, Nnenna Henrietta Umoke, Pelumi Adanigbo, Rukayat Olajumoke Babatunde, Abisoye Oyebisola Fafioye, Ruth Joseph Adara, Emmanuella Amara Ofoka, Kelechi Purity Ezennubia, Oshoma Erumiseli, Ikhazuagbe Hilary Ifijen

**Affiliations:** a Department of Pure and Industrial Chemistry, Nnamdi Azikiwe University Awka Nigeria; b College of Health Sciences, Ebonyi State University Nigeria; c Department of Chemistry and Biochemistry, George Mason University Fairfax VA USA; d Department of Biochemistry, Bayero University Kano PMB 3011 Nigeria; e Department of Pharmacy 1 Queen Elizabeth Road Ibadan Oyo Nigeria; f Western Kentucky University, 1906 College Heights Blvd Bowling Green KY 42101 USA; g Chemistry and Biochemistry Department, Baylor University 1311 S 5th St Waco Texas Tx. 76706 USA; h Department of Chemistry, Oregon State University 97330 USA; i Department of Research Outreach, Rubber Research Institute of Nigeria Benin City Edo State Nigeria larylans4u@yahoo.com

## Abstract

Cancer remains a global health burden, with conventional treatment strategies such as chemotherapy and radiotherapy often constrained by systemic toxicity, therapeutic resistance, and suboptimal tumor eradication. The development of synergistic treatment modalities is essential to enhance efficacy while minimizing adverse effects. Radiotherapy-chemodynamic therapy (RT-CDT) has emerged as a promising approach that couples the DNA-damaging power of ionizing radiation with the oxidative stress induced by chemodynamic reactions in the tumor microenvironment. Central to this strategy are bismuth-based nanoparticles (BiNPs), which serve as both potent radiosensitizers and catalytic agents for reactive oxygen species (ROS) generation due to their high atomic number, robust X-ray absorption, and favorable physicochemical and biocompatibility profiles. This review explores the fundamental mechanisms through which BiNPs enhance RT and CDT efficacy, including their roles in secondary electron generation, ROS amplification, and DNA damage. Various bismuth nanoplatforms—such as bismuth oxide, bismuth sulfide, and bismuth vanadate—are discussed with respect to their structural attributes, catalytic activity, and tumor-targeting capacities. Emphasis is placed on the design and engineering of multifunctional, surface-modified, and hybrid BiNP systems that enable combinatory therapeutic action and real-time monitoring *via* dual-modality imaging, including computed tomography (CT) and photoacoustic imaging. Preclinical studies demonstrate that BiNP-based RT-CDT significantly inhibits tumor progression, validating their potential in enhancing radiotherapeutic outcomes. Nonetheless, translational challenges persist, including nanoparticle cytotoxicity, *in vivo* stability, large-scale production, and regulatory hurdles. Addressing these limitations through rational design and safety optimization is critical for clinical application. Looking ahead, the integration of BiNPs into image-guided RT-CDT platforms presents a compelling opportunity for more targeted, efficient, and minimally invasive cancer therapies.

## Abstract 2

Cancer remains a global health burden, with conventional treatment strategies such as chemotherapy and radiotherapy often constrained by systemic toxicity, therapeutic resistance, and suboptimal tumor eradication.

## Graphical abstract

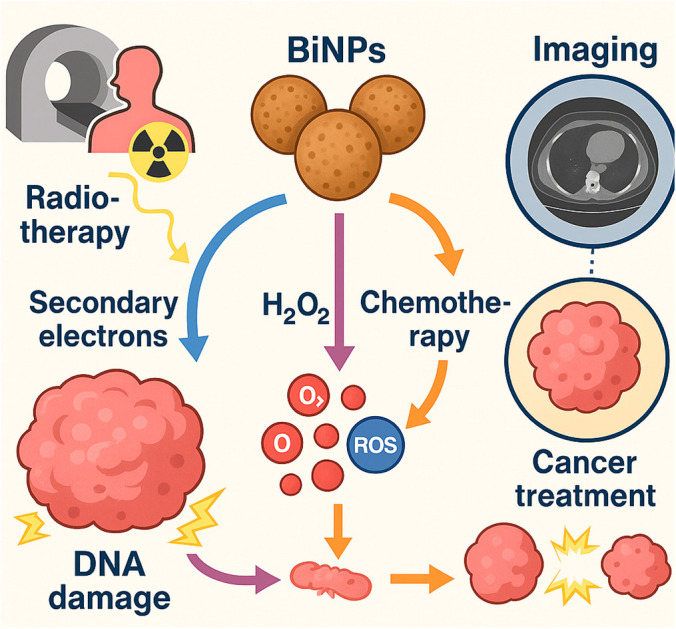

## 1 Introduction

Cancer remains a leading cause of morbidity and mortality worldwide, accounting for millions of deaths annually despite decades of research and clinical advancement. Traditional cancer treatment modalities—including surgical resection, chemotherapy, and radiotherapy—constitute the cornerstone of oncological care.^1–3^ However, these conventional approaches are frequently associated with significant limitations. Surgical interventions are often invasive and constrained by tumor accessibility and the risk of recurrence. Chemotherapy, although systemically effective, suffers from poor tumor selectivity, leading to extensive off-target toxicity and adverse side effects.^4–9^ Radiotherapy (RT), which employs ionizing radiation to induce DNA strand breaks and apoptosis in malignant cells, is a mainstay in clinical oncology but is also hindered by inherent challenges such as the development of radioresistance in tumor cells and the collateral damage inflicted upon adjacent normal tissues.^10,11^

In recent years, there has been a paradigm shift toward more targeted and tumor-specific therapeutic strategies, with nanomedicine and tumor microenvironment (TME)-responsive therapies gaining considerable attention.^12,13^ One such emerging strategy is chemodynamic therapy (CDT), a non-invasive modality that exploits the aberrant biochemical characteristics of the TME—such as elevated levels of hydrogen peroxide (H_2_O_2_), acidic pH, and glutathione (GSH)—to initiate localized Fenton or Fenton-like reactions. These reactions catalyze the *in situ* generation of highly reactive hydroxyl radicals (˙OH), leading to oxidative damage of intracellular components and selective tumor cell apoptosis without requiring external energy input. The self-sustained and tumor-specific nature of CDT offers a significant therapeutic advantage, particularly in hypoxic or drug-resistant tumor regions.^14,15^

Recognizing the complementary mechanisms of RT and CDT, their integration into a unified treatment paradigm—Radiotherapy-chemodynamic therapy (RT-CDT)—has emerged as a promising synergistic approach. RT-CDT combines the DNA-damaging capabilities of ionizing radiation with the oxidative stress-inducing power of CDT, thereby enhancing reactive oxygen species (ROS) accumulation and maximizing cytotoxic efficacy. This combination not only potentiates the therapeutic outcome but also allows for reduced dosages of radiation and chemotherapeutic agents, thereby mitigating systemic toxicity and improving patient tolerability.^16–18^

Among the various nanomaterials developed for RT-CDT applications, bismuth-based nanoparticles (BiNPs) have gained particular prominence due to their unique physicochemical properties. Bismuth (atomic number *Z* = 83) possesses a high atomic mass, which enables efficient X-ray absorption and energy deposition, making BiNPs excellent radiosensitizers that can significantly amplify the effects of radiotherapy.^19,20^ Additionally, BiNPs exhibit favorable biocompatibility, low toxicity, and surface modifiability, which are critical attributes for *in vivo* biomedical applications. Recent studies have also demonstrated their potential catalytic activity in Fenton-like reactions, positioning them as dual-function agents capable of enhancing both RT and CDT. Furthermore, the ability to functionalize BiNPs with targeting ligands, imaging moieties, or therapeutic payloads enhances their versatility as platforms for precision nanomedicine.^21,22^

While bismuth-based nanoparticles (BiNPs) have been widely reviewed for their applications in photothermal therapy (PTT) and photodynamic therapy (PDT), significantly less attention has been given to their emerging role in radiotherapy-chemodynamic therapy (RT-CDT). RT-CDT represents a synergistic approach that leverages the DNA-damaging effects of ionizing radiation alongside Fenton-like catalytic reactions to generate cytotoxic reactive oxygen species (ROS) within the tumor microenvironment. Unlike prior reviews that broadly cover BiNPs in various therapeutic contexts, this review uniquely centers on their dual function as radiosensitizers and chemodynamic catalysts within RT-CDT systems. By narrowing the scope to this promising combinatory strategy, we provide an in-depth exploration of the physicochemical design, functional mechanisms, and therapeutic outcomes of BiNP-based nanoplatforms tailored for RT-CDT, thus addressing a critical gap in current nanomedicine literature.

The synthesis of bismuth nanoparticles is not the focus of this review. Instead, emphasis is placed on the urgent need for BiNPs possessing well-defined and significant functional attributes—such as catalytic efficiency, tumor selectivity, and biocompatibility—tailored to meet the requirements of RT-CDT applications. By prioritizing structure–function relationships and biological performance over synthetic routes, the review highlights the translational potential of these nanostructures in advancing next-generation cancer therapies.

This review provides an in-depth analysis of the recent advancements in bismuth-based nanoplatforms for radiotherapy-chemodynamic therapy (RT-CDT), emphasizing their dual functionality in enhancing radiation dose deposition and catalyzing reactive oxygen species generation. It explores the underlying mechanisms of action, structural and surface modifications, and preclinical therapeutic outcomes associated with these nanomaterials. By integrating diagnostic and therapeutic capabilities, bismuth nanoparticles offer a powerful approach to overcoming the inherent limitations of conventional cancer treatments. Their application in RT-CDT underscores a critical advancement toward the development of safe, effective, and clinically viable multifunctional agents for precision cancer therapy.

## 2 Mechanisms of RT-CDT with bismuth-based nanoparticles

### 2.1 Radiotherapy enhancement by bismuth

Bismuth (Bi), with its high atomic number (*Z* = 83), plays a pivotal role in enhancing the efficacy of radiotherapy through multiple interrelated mechanisms. Its inherent physical and chemical properties make bismuth-based nanoparticles (BiNPs) highly effective radiosensitizers, capable of increasing localized radiation dose deposition and augmenting oxidative stress within the tumor microenvironment (TME).^23–25^ The radiotherapy enhancement mechanisms associated with BiNPs include.

#### 2.1.1 High X-ray absorption coefficient

Radiotherapy (RT) remains a mainstay in the treatment of numerous malignancies due to its capacity to induce irreparable DNA damage in tumor cells *via* ionizing radiation. Despite its broad clinical use, several inherent limitations—including poor selectivity, suboptimal energy deposition, and damage to adjacent healthy tissues—constrain its therapeutic index.^26^ Consequently, improving the tumor-specific efficacy of RT while minimizing its systemic toxicity has become a major focus of ongoing research. Among the strategies developed to overcome these limitations, the incorporation of nanotechnology into RT protocols has shown remarkable promise, particularly through the use of high atomic number (high-*Z*) nanomaterials as radiosensitizers.^27,28^

Bismuth (*Z* = 83), owing to its elevated atomic number and favorable safety profile compared to other heavy metals, offers significant advantages in radiotherapy applications. Due to its high atomic number, bismuth exhibits exceptional X-ray attenuation capabilities, surpassing many conventionally used materials such as iodine or gold. This high attenuation is primarily a consequence of the photoelectric effect, which dominates X-ray interactions at kiloelectronvolt (keV) energy levels typically utilized in clinical radiotherapy settings. Since the probability of photoelectric absorption is proportional to *Z*^3^ and inversely proportional to the photon energy cubed (1/*E*^3^), bismuth-based nanoparticles (BiNPs) can markedly enhance the absorption of ionizing radiation within tumor sites.^29,30^ This results in the amplification of localized energy deposition, thereby boosting the radiolytic generation of secondary electrons and reactive oxygen species (ROS), which contribute directly to DNA double-strand breaks and apoptosis in cancer cells.^31^

The work of Kavousi *et al.* (2024) highlights the growing recognition of bismuth-based nanomedicine as a transformative platform for overcoming long-standing challenges in RT, such as radioresistance, hypoxia, and collateral toxicity.^22^ Their review underscores the versatility of BiNPs—not only in enhancing radiation-induced cytotoxicity but also in enabling multimodal theranostics, which combine imaging and therapy functionalities within a single nanosystem. According to their findings, BiNPs can be synthesized in various morphologies (*e.g.*, core–shell, hollow, hybrid) and functionalized to exhibit near-infrared (NIR) absorbance, photothermal conversion efficiency, prolonged systemic circulation, and tumor-selective accumulation. These attributes collectively contribute to improved radiosensitization and therapeutic outcomes.

**Fig. 1:**
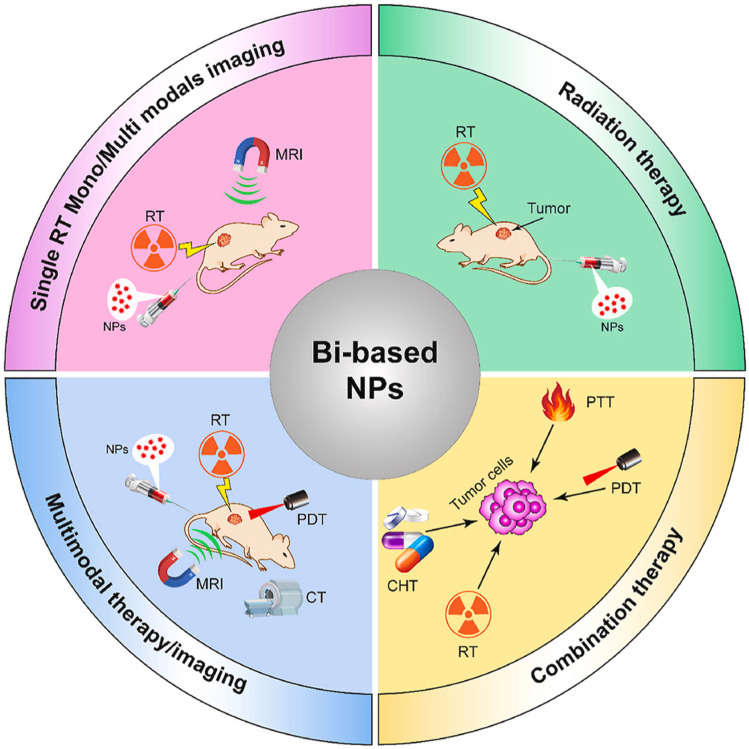
Schematic illustration of a smart bismuth-based nanoplatform designed for radiotherapy enhancement and multimodal cancer therapy.^22^

Furthermore, the integration of BiNPs into smart nanoplatforms (as illustrated in Fig. 1) has facilitated the development of synergistic treatment systems that merge RT with other modalities such as chemodynamic therapy (CDT), photothermal therapy (PTT), and immunotherapy. These multifunctional platforms are specifically engineered to exploit the tumor microenvironment's pathological features—such as acidic pH, elevated H_2_O_2_ levels, and aberrant vasculature—to achieve site-specific activation, thereby maximizing therapeutic efficacy while minimizing harm to healthy tissues. The inclusion of bismuth-based nanomaterials in RT protocols exemplifies a promising paradigm shift in cancer therapy. By harnessing bismuth's intrinsic physical and chemical advantages, researchers are paving the way toward precision radiotherapy strategies that are both highly effective and clinically adaptable.

Building upon these foundational mechanisms, Kavousi *et al.* (2024) highlight the transformative potential of bismuth-based nanoparticles (BiNPs) in radiotherapy.^22^ They argue that while RT remains a cornerstone in oncological treatment, its limitations—such as tumor hypoxia, radioresistance, and collateral tissue damage—necessitate innovative approaches. BiNPs, due to their high X-ray attenuation, NIR absorbance, and long systemic circulation, serve as multifunctional platforms that address these challenges. Kavousi *et al.*^22^ provide an overview of BiNPs' utility in theranostics, integrating diagnostics and therapy to overcome spatial and biological barriers that undermine RT outcomes. Their review points to the growing potential of BiNPs in multimodal synergistic treatments, encompassing radiotherapy, photothermal therapy (PTT), and imaging-guided interventions.

Extending these insights, Du *et al.* (2017) offer a compelling demonstration of the targeted radiosensitization and imaging capabislity of BiNPs through the synthesis of hyaluronic acid-functionalized Bi_2_O_3_ nanoparticles (HA-Bi_2_O_3_ NPs).^32^ Using a one-pot hydrothermal method, they created nanoparticles with improved aqueous solubility, enhanced biocompatibility, and specific tumor targeting *via* CD44 receptor-mediated uptake. These HA-Bi_2_O_3_ NPs not only maintained high X-ray attenuation efficiency but also demonstrated potent dose-dependent radiosensitization *in vitro* by synergizing with X-rays to induce apoptosis and arrest the cell cycle. Notably, these particles enabled active-targeted CT imaging, thereby exemplifying the theranostic integration of bismuth nanostructures for diagnosis and real-time image-guided radiotherapy. The targeted delivery system employed by Du *et al.*^32^ not only improves tumor specificity but also reduces off-target toxicity—two key challenges in clinical radiotherapy.

In parallel, Wen *et al.* (2022) investigated the design strategies that optimize the performance of BiNPs.^33^ While acknowledging the intrinsic radiophysical advantages of bismuth, they emphasize that pure BiNPs may fall short due to poor tumor retention and long-term toxicity risks.^33^ To address these, surface modification—like the hyaluronic acid functionalization noted by Du *et al.—*is cited as essential for enhancing biocompatibility and tumor targeting.^32^ Wen *et al.*^33^ further explore elemental co-doping (*e.g.*, Fe^3+^) to enable multimodal imaging and structural modifications to improve chemotherapeutic loading and biodistribution. Such engineered BiNPs are also shown to be amenable to combination therapies (*e.g.*, PTT and HIFU), reinforcing their utility as part of comprehensive oncologic regimens.

While the therapeutic role of BiNPs continues to evolve, Saad *et al.* (2022) present a complementary approach by leveraging bismuth's radioprotective capacity.^34^ They designed bismuth-based fusible alloys (Bi–Pb–Sn and Bi–Pb–Sn–Cd) as custom-molded shielding blocks to protect healthy tissues during radiotherapy. Their work confirmed that cadmium-free Bi alloys not only possess excellent attenuation coefficients and Vickers microhardness but also contribute to eco-friendly clinical practices. The presence of a novel Pb_7_Bi_3_ metastable crystalline phase was associated with enhanced mechanical strength, making these materials suitable for clinical-grade shielding with precise patient conformation.

Taken together, the findings of Kavousi *et al.* (2024),^22^ Du *et al.* (2017),^32^ Wen *et al.* (2022),^33^ and Saad *et al.* (2022) collectively underscore the multifunctional promise of bismuth-based nanomaterials in radiotherapeutic paradigms.^34^ Whether functioning as tumor-targeted radiosensitizers,^32,33^ theranostic agents,^22^ or radiation-shielding materials,^34^ bismuth's high atomic number enables significant advances in localized dose enhancement and treatment specificity. The shared thread across all studies is the capacity of bismuth to amplify the photoelectric effect, thereby increasing radiation energy deposition precisely within tumor sites while minimizing systemic burden. These findings chart a forward-looking path toward precision oncology, where BiNPs are integral to image-guided, minimally invasive, and synergistic cancer therapies.

#### 2.1.2 Increased radiation dose deposition

The concept of dose-enhancement through nanoparticle-mediated radiosensitization has garnered considerable interest as a means to address the dual challenges of tumor radioresistance and collateral damage to healthy tissues during radiotherapy. Bismuth-based nanoparticles (BiNPs), owing to their high atomic number (*Z* = 83), present a superior alternative to conventional radiosensitizers such as gold nanoparticles (AuNPs) for enhancing radiation deposition selectively within tumor microenvironments.^35,36^ Their capacity to accumulate preferentially within malignant tissues and amplify local radiation effects forms the basis of the dose-enhancement effect, which improves treatment efficacy while limiting systemic toxicity.^37^

In a pivotal study, Farahani *et al.* (2020) quantitatively evaluated the radiosensitization performance of BiNPs in comparison to AuNPs under clinically relevant radiation energies.^38^ Utilizing nanoparticle-impregnated polymer gel dosimeters, the study demonstrated that BiNPs achieved a greater dose enhancement factor (DEF) of 16.35% under iridium-192 (380 keV) brachytherapy, surpassing the DEF observed for AuNPs (14.72%) at equivalent concentrations. This performance advantage is attributed to the higher atomic number of bismuth, which increases photoelectric interaction probability, particularly under low-energy photon exposure. In contrast, under higher-energy cobalt-60 gamma irradiation (1.25 MeV), both nanoparticles exhibited DEFs below 4%, underscoring the energy-dependent nature of nanoparticle-mediated dose enhancement.

These findings are particularly significant when viewed through the lens of selective tumor radiosensitization. Because BiNPs preferentially accumulate in tumor tissues—either passively *via* the enhanced permeability and retention (EPR) effect or actively through ligand-mediated targeting—the radiation amplification remains confined to the tumor. This spatial specificity translates to heightened radiation-induced cytotoxicity in cancerous cells, while sparing surrounding normal tissues. Farahani *et al.*‘s data therefore affirm the feasibility of utilizing BiNPs to improve the therapeutic index of radiotherapy by exploiting tumor-specific nanoparticle localization.^38^

Further innovation in this domain is exemplified by Bai *et al.* (2022), who designed a sophisticated bismuth-based theranostic nanoplatform—TPP-Bi@PDA@CP—capable of two-stage targeting to hepatocellular carcinoma cells and mitochondria.^39^ This system achieved tumor accumulation 2.63 times greater than that of conventional EPR-mediated delivery, significantly enhancing site-specific radiosensitization. In addition, the nanoplatform displayed superior CT contrast enhancement (∼51.8 HU mL mg^−1^) and photothermal conversion efficiency (52.3%), enabling real-time imaging and multimodal therapy. Impressively, tumor inhibition was achieved at remarkably low doses (15–25 μg mL^−1^), thereby reducing systemic toxicity and highlighting the clinical viability of low-dose, high-efficacy radiotherapeutic strategies.

The success of Bai *et al.*'s system lies in its synergistic design, integrating targeted delivery, image guidance, and photothermal therapy, alongside its radiosensitization capability.^39^ Importantly, the incorporation of an immune-enhancing polysaccharide coating facilitated post-treatment immune response activation, contributing to tumor recurrence inhibition—a feature critical for long-term clinical outcomes.

Collectively, the evidence from Farahani *et al.* (2020)^38^ and Bai *et al.* (2022)^39^ converges to substantiate the mechanistic principle that BiNPs, when localized in tumors, induce a selective increase in radiation dose, thereby enhancing tumor cell kill while minimizing harm to adjacent normal tissue. These results underscore the importance of not only selecting high-*Z* materials like bismuth for radiosensitization but also engineering delivery systems that maximize tumor-specific accumulation and optimize radiation energy compatibility. BiNPs offer a strategically advantageous platform for enhancing the precision and efficacy of radiotherapy. Their capacity to amplify radiation selectively within tumors makes them promising candidates for next-generation nanoparticle-assisted radiotherapy protocols, particularly in conjunction with brachytherapy or other low-energy radiation modalities. Future work should focus on clinical translation, long-term biocompatibility, and integration with emerging modalities such as immunoradiotherapy and real-time image-guided treatment.

#### 2.1.3 Generation of secondary electrons

The clinical utility of bismuth nanoparticles (BiNPs) as radiosensitizers in cancer therapy is underpinned by their unique physicochemical characteristics, particularly their high atomic number (*Z* = 83), which enables effective interaction with ionizing radiation such as X-rays and gamma rays.^40–42^ Upon irradiation, BiNPs absorb high-energy photons and emit a cascade of secondary electrons—including photoelectrons, Auger electrons, and Compton-scattered electrons. These high-energy particles can directly ionize biomolecules within the tumor microenvironment and, more importantly, initiate radiolysis of surrounding water molecules, leading to the formation of cytotoxic reactive oxygen species (ROS), such as hydroxyl radicals (˙OH). These ROS inflict extensive oxidative damage on cellular components including DNA, proteins, and lipids, thereby amplifying radiation-induced cell death with heightened precision and efficacy.^43,44^

However, one of the major challenges facing the clinical translation of BiNPs lies in their long-term biocompatibility and potential for accumulation in off-target tissues. Addressing this concern, Deng *et al.* (2018) developed folate-functionalized, red blood cell (RBC) membrane-coated bismuth nanoparticles (F-RBC BiNPs), which exhibit a biodegradable architecture designed for enhanced tumor selectivity and safe systemic clearance.^45^ The RBC membrane coating imparts immune-evasive “stealth” properties, prolonging circulation time, while the conjugated folate moieties enable receptor-mediated targeting of tumor cells that overexpress folate receptors, such as in breast cancer. This targeted delivery ensures localized accumulation of BiNPs at the tumor site, where they can effectively potentiate radiotherapy by catalyzing the generation of secondary electrons and ROS.

**Fig. 2:**
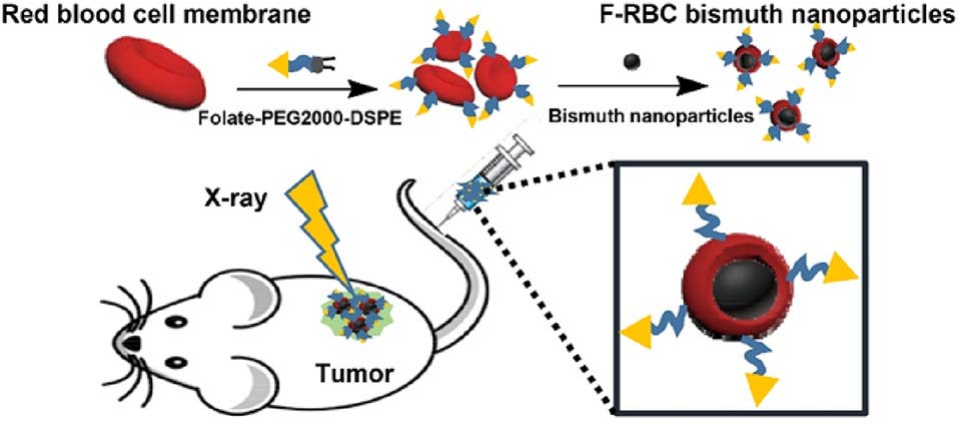
Tumor-targeted, biodegradable, and stealth bismuth nanoparticles for enhanced X-ray radiotherapy in breast cancer treatment.^45^

*In vivo* studies using breast cancer-bearing mice revealed significant tumor inhibition and prolonged survival when X-ray therapy was combined with F-RBC BiNPs. Notably, post-treatment biodistribution analyses indicated that these nanoparticles were bioresorbed and eliminated from the body within 15 days, with no observable histopathological damage or inflammation in vital organs. This degradability addresses a key translational barrier, minimizing long-term toxicity while maintaining therapeutic potency. The concept is further illustrated in Fig. 2, which depicts a schematic model of tumor-targeted, biodegradable, and stealth-coated BiNPs engineered for enhanced X-ray radiotherapy. The figure encapsulates the design strategy and mechanistic action, highlighting how the synergistic combination of targeting, biocompatibility, and radiosensitization can be harnessed to improve therapeutic outcomes in breast cancer treatment.

Jiao *et al.* (2018) further tackled the critical challenge of creating biocompatible, radiotherapy-enhancing, and imaging-capable nanomaterials by synthesizing cellulose nanofiber-templated ultra-small bismuth nanoparticles (BiNPs) with precisely tunable sizes between 2 and 10 nm.^46^ The synthesis strategy leveraged the interstitial spaces within functionalized cellulose nanofibers, which served as nanoreactors and stabilizers for bismuth ion reduction. This green, template-assisted approach allowed precise control over nanoparticle dimensions and surface carboxylation, facilitating high aqueous stability and potentially favorable renal clearance profiles. Importantly, these ultrasmall BiNPs exhibited strong X-ray attenuation capacity and high levels of ROS generation upon irradiation, particularly hydroxyl radicals (˙OH), which are potent inducers of DNA and cellular damage. *In vitro* cytotoxicity studies and *in vivo* experiments using tumor-bearing mice demonstrated that these BiNPs significantly enhanced radiation-induced tumor growth inhibition. Their concentration-dependent cytotoxicity was closely correlated with ROS production, substantiating their mechanistic action through secondary electron generation and radiolytic ROS-mediated cellular disruption.

Expanding the functionality of BiNPs beyond radiosensitization, Peng *et al.* (2025) introduced a class of bismuth-loaded poly(α-amino acid) nanoparticles (Bi@POS) engineered for bimodal X-ray computed tomography (CT) and fluorescence imaging (FI).^47^ This dual-imaging capability is a highly desirable feature in precision nanomedicine, enabling both deep-tissue anatomical visualization *via* CT and high-sensitivity molecular imaging through FI. The incorporation of fluorescent dyes—FITC and ICG—into the BiNP matrix endowed the particles with bright and stable fluorescence emission, while the bismuth core maintained excellent CT contrast performance. Notably, the catechol-containing polymeric shell not only enabled efficient Bi^3+^ chelation but also imparted reactive oxygen and nitrogen species (RONS) scavenging activity, offering additional protection to healthy tissues and reducing oxidative stress in non-targeted areas.

The aforementioned studies exemplify a new generation of BiNP-based nanotherapeutics that are simultaneously biocompatible, radiotherapy-enhancing, and imaging-capable. Jiao *et al.* emphasize the value of size-controlled, biodegradable radiosensitizers tailored for efficient clearance and tumor-selective damage, while Peng *et al.* highlight the translational advantage of combining therapeutic radiosensitization with diagnostic imaging, forming a basis for image-guided and personalized cancer therapy. These complementary findings underscore the versatile platform potential of BiNPs, especially when designed with careful consideration of size, surface functionality, and multimodal capability. Going forward, integrating degradable, ROS-generating BiNPs with imaging features such as CT and FI can enable real-time monitoring of therapeutic response, improve targeting precision, and reduce systemic toxicity—key goals in advancing the next generation of smart, nanostructured radiosensitizers for oncological applications.

#### 2.1.4 ROS amplification and DNA damage

The elevated production of reactive oxygen species (ROS) mediated by bismuth-based nanoparticles (BiNPs) plays a crucial role in intensifying oxidative stress within cancer cells, thereby significantly enhancing the effectiveness of radiotherapy. When exposed to ionizing radiation such as X-rays, BiNPs demonstrate superior X-ray absorption due to bismuth's high atomic number (*Z* = 83), which enhances localized energy deposition in tumor tissues 20.

Upon irradiation, BiNPs emit a cascade of secondary electrons—including photoelectrons, Auger electrons, and Compton electrons—that initiate radiolysis of intracellular water, resulting in the production of highly reactive species, particularly hydroxyl radicals (˙OH). These ROS are central to the mechanism of radiotherapy-chemodynamic therapy (RT-CDT), as they cause irreversible oxidative damage to critical biomolecules such as lipids, proteins, and nucleic acids. This includes the induction of mitochondrial dysfunction and DNA double-strand breaks (DSBs)—a lethal form of DNA damage that often leads to apoptosis or cell cycle arrest.^20,44^

Importantly, BiNPs exhibit a degree of selectivity in targeting tumor cells while sparing healthy tissues, a feature rooted in the biological and biochemical differences between cancerous and normal cells. Cancer cells inherently maintain elevated basal ROS levels due to accelerated metabolism, mitochondrial dysregulation, and sustained proliferation. However, they also possess weakened antioxidant defense systems, including reduced levels of glutathione (GSH), superoxide dismutase (SOD), and catalase. As a result, when ROS levels are further elevated by BiNP-mediated therapy, cancer cells are pushed beyond their oxidative stress threshold, leading to extensive biomolecular damage, including localized DNA fragmentation, and ultimately, cell death.

In contrast, healthy cells have more efficient antioxidant systems, enabling them to detoxify ROS more effectively and maintain redox homeostasis. This biochemical resilience protects non-malignant tissues from the ROS-induced damage experienced by cancer cells during BiNP-facilitated RT-CDT. Furthermore, the acidic and hypoxic tumor microenvironment enhances the selectivity of BiNP activity. In such environments, especially when BiNPs are doped with transition metals, Fenton-like or pseudo-Fenton reactions are triggered, which selectively generate additional ˙OH radicals. These reactions are significantly less active in the neutral pH of healthy tissues, making the oxidative assault highly localized to tumor sites.^20,44^

Additionally, the enhanced permeability and retention (EPR) effect observed in solid tumors allows for preferential accumulation and retention of BiNPs in cancerous tissue, further reinforcing their tumor-specific action. This passive targeting mechanism enables more effective ROS-mediated cytotoxicity within the tumor mass, while limiting exposure to surrounding normal tissue. The selective induction of localized DNA damage in cancer cells by BiNPs arises from a combination of physical properties (*e.g.*, high-*Z* for radiosensitization), biological selectivity (*e.g.*, differential antioxidant capacity), and tumor microenvironmental factors (*e.g.*, acidity and hypoxia). These synergistic features ensure that oxidative stress is concentrated within tumor cells, leading to targeted cytotoxicity while minimizing collateral damage to healthy tissues. As such, BiNPs hold substantial promise as next generation radiosensitizers and chemodynamic agents in precision oncology.

In line with this mechanism, Ahamed *et al.* (2019) investigated the cytotoxic and pro-apoptotic effects of Bi_2_O_3_ nanoparticles in MCF-7 human breast cancer cells.^48^ Their findings clearly demonstrated that Bi_2_O_3_ NPs significantly reduced cell viability and disrupted membrane integrity in a dose-dependent manner. Notably, the nanoparticles induced oxidative stress characterized by elevated ROS levels, increased lipid peroxidation, depletion of intracellular glutathione (GSH), and suppressed superoxide dismutase (SOD) activity. This redox imbalance led to DNA damage and apoptosis *via* the Bax/Bcl-2/caspase-3 pathway, further confirming the mechanistic role of ROS in mediating the cytotoxic effects. The reversal of these effects upon *N*-acetylcysteine (NAC) supplementation underscored the centrality of oxidative stress in BiNP-induced cytotoxicity. Collectively, these results affirm the capacity of BiNPs to enhance ROS-mediated radiotherapy responses through redox modulation and apoptotic signaling.

However, the potential of BiNPs in radiosensitization is also influenced by their structural stability under irradiation.

Expanding on the multifunctional potential of BiNPs, Oztas *et al.* (2019) developed a novel theranostic platform using ultrasmall BiNPs (3.6 nm) conjugated with the tumor-penetrating peptide LyP-1.^49^ These Bi-LyP-1 NPs exhibited targeted tumor accumulation and were capable of dual-modal imaging—computed tomography (CT) and photoacoustic (PA)—due to their efficient absorption in the ionizing radiation and second near-infrared (NIR-II) spectral regions. The integration of photothermal therapy (PTT) with radiotherapy significantly elevated the therapeutic efficiency by promoting further ROS generation and localized hyperthermia, which disrupts cancer cell homeostasis and sensitizes tumors to radiation. Remarkably, these nanoparticles were rapidly cleared through renal and fecal pathways, minimizing concerns of long-term toxicity and accumulation. This study exemplifies how BiNPs can be engineered not only for enhanced radiosensitization *via* ROS production but also for imaging-guided, multi-modal cancer treatment with favorable pharmacokinetics.

The collective findings from these studies underscore the profound role of BiNP-induced oxidative stress in enhancing radiotherapy outcomes. By amplifying ROS-mediated DNA and mitochondrial damage, improving radiation dose localization, and enabling multi-modal treatment strategies, BiNPs emerge as a versatile and potent class of radiosensitizers. Their continued development holds significant promise for advancing RT-CDT platforms toward more effective and safer cancer therapies.

### 2.2 Chemodynamic therapy *via* Fenton-like reactions

Chemodynamic therapy (CDT) represents a promising cancer treatment approach that leverages the unique tumor microenvironment to selectively generate toxic reactive oxygen species (ROS) *in situ*, leading to targeted cancer cell death. Among the various materials explored for CDT, bismuth compounds such as bismuth oxide (Bi_2_O_3_) and bismuth vanadate (BiVO_4_) have garnered significant attention due to their ability to catalyze ROS production through Fenton-like reactions specifically within the acidic milieu characteristic of tumor tissues.^50–52^ These compounds take advantage of the slightly acidic pH often found in tumors (typically around pH 6.5 or lower), which facilitates the catalytic conversion of endogenous hydrogen peroxide (H_2_O_2_)—a molecule naturally overexpressed in many cancer cells—into highly reactive hydroxyl radicals (˙OH).^52^

The generation of hydroxyl radicals through these Fenton-like reactions is a key mechanism by which bismuth compounds exert their therapeutic effects in CDT. Hydroxyl radicals are among the most potent ROS due to their high reactivity and ability to damage a wide array of biomolecules, including DNA, proteins, and lipids. When Bi_2_O_3_ or BiVO_4_ nanoparticles enter the tumor microenvironment, they interact with the elevated concentrations of H_2_O_2_ present, catalyzing its decomposition into ˙OH radicals.^41,51^ This localized surge in ROS production leads to oxidative stress that exceeds the antioxidant defense capacity of cancer cells, thereby disrupting cellular redox homeostasis. The resulting oxidative damage initiates signaling cascades that culminate in programmed cell death pathways such as apoptosis, characterized by DNA fragmentation, mitochondrial membrane potential disruption, and caspase activation. Additionally, the oxidative assault can trigger ferroptosis, a form of regulated cell death distinct from apoptosis, which is driven by iron-dependent lipid peroxidation and accumulation of ROS within cellular membranes.^52,53^

By confining ROS generation to the tumor site, bismuth-based CDT minimizes damage to surrounding healthy tissues, thereby reducing systemic toxicity. This selectivity is crucial for improving the therapeutic index of cancer treatments. Moreover, the dual capability of bismuth compounds to catalyze ROS formation and induce multiple cell death pathways not only enhances the overall cytotoxicity against cancer cells but also helps overcome resistance mechanisms that tumors often develop against conventional therapies. Consequently, the utilization of bismuth compounds in CDT through Fenton-like reactions offers a powerful strategy to harness endogenous biochemical features of tumors for targeted oxidative damage, ultimately advancing the efficacy and precision of cancer treatment modalities.

For example, Tang *et al.* (2025) introduce a pioneering approach to cancer immunotherapy through a pentavalent bismuth(v)-based nanoplatform, NaBiVO_3_-PEG, that overcomes common barriers associated with traditional ROS-mediated therapies.^54^ Unlike conventional systems that depend heavily on exogenous excitation sources or the presence of endogenous H_2_O_2_ and O_2_ to generate reactive oxygen species (ROS), this system leverages an intrinsic, stimulus-independent mechanism. Specifically, the nanoplatform capitalizes on the acidic tumor microenvironment to initiate a H^+^-accelerated hydrolysis, which induces spontaneous generation of hydroxyl radicals (˙OH) and singlet oxygen (^1^O_2_). These ROS are generated through a Bi^5+^ to Bi^3+^ electron transfer coupled with lattice oxygen transformation, facilitating potent oxidative stress directly within tumor cells.

**Fig. 3:**
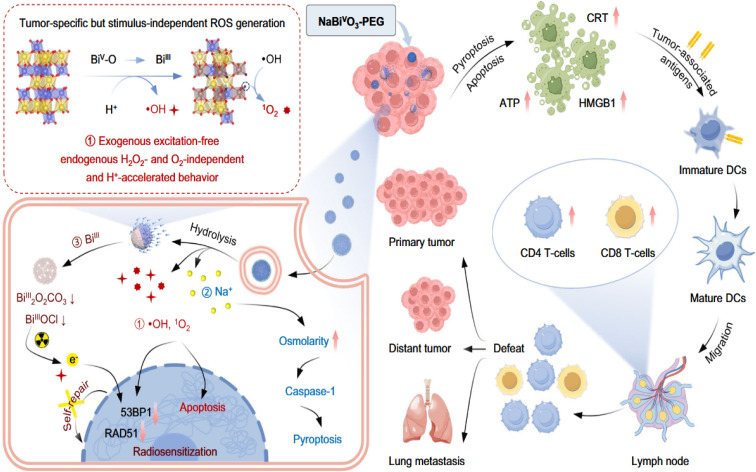
Schematic illustration of NaBiVO_3_-PEG nanoplatform for tumor immunotherapy, functioning independently of exogenous excitation, H_2_O_2_, and O_2_.^54^

The figure (Fig. 3) provides a comprehensive visual of this mechanism. Once NaBiVO_3_-PEG accumulates at the tumor site or is endocytosed by tumor cells, it hydrolyzes in the acidic environment, not only initiating ROS production but also releasing Na^+^ ions. This ionic shift increases cellular osmolarity and activates caspase-1, promoting pyroptosis—an inflammatory form of programmed cell death—alongside apoptosis. Together, these effects lead to tumor cell death while simultaneously triggering immunogenic responses.

Additionally, insoluble BiVO_4_ and Bi_2_O_3_ hydrolysates form *in situ*, contributing to optional radiosensitization by facilitating further ROS generation under ionizing radiation and disrupting DNA repair pathways (*e.g.*, *via* 53BP1 and RAD51 suppression). These features render NaBiVO_3_-PEG effective not only as a stand-alone therapeutic but also as a potent radiosensitizer for combinatorial radio-immunotherapy.

Critically, as illustrated in the figure, dying tumor cells undergoing apoptosis and pyroptosis release damage-associated molecular patterns (DAMPs) such as CRT, ATP, and HMGB1, which serve as “danger signals” that recruit and activate dendritic cells (DCs). This leads to the maturation of immature DCs and subsequent activation of CD4^+^ and CD8^+^ T-cells, fostering a systemic immune response. These immunological changes enable the body to recognize and attack not only the primary tumor but also distant metastatic lesions, including lung metastases—an outcome graphically represented by tumor defeat and immune cell migration in the lymph nodes.

This study underscores the utility of NaBiVO_3_-PEG as a multifunctional immunotherapeutic nanoplatform that bypasses limitations of oxygen and H_2_O_2_ dependency, while simultaneously enhancing radiotherapy, activating immune pathways, and offering imaging capabilities *via* CT. The integration of biochemical self-activation, ROS-based cytotoxicity, and immune stimulation makes it a strong candidate for next-generation oncologic nanomedicine.

## 3 Bismuth-based nanoparticle platforms for RT-CDT

### 3.1 Types of bismuth nanomaterials

#### 3.1.1 Bismuth oxide (Bi_2_O_3_) nanoparticles: excellent radiosensitizers with CDT potential

Bismuth oxide (Bi_2_O_3_) nanoparticles have emerged as excellent radiosensitizers with remarkable potential in cancer diagnosis and therapy (CDT) owing to their distinctive physicochemical characteristics. The primary attribute that sets Bi_2_O_3_ nanoparticles apart is their high atomic number (*Z* = 83), which plays a pivotal role in enhancing their interaction with ionizing radiation. Materials with high atomic numbers have a stronger ability to absorb and scatter X-rays through photoelectric and Compton interactions. In the context of radiotherapy, this means that Bi_2_O_3_ nanoparticles can significantly amplify the local radiation dose within tumor tissues, leading to enhanced tumor cell damage while sparing surrounding healthy tissues.^55,56^

The high X-ray absorption coefficient of Bi_2_O_3_ further supports its role as a potent radiosensitizer. When irradiated, Bi_2_O_3_ nanoparticles produce a cascade of secondary electrons, including Auger and photoelectrons, which induce localized oxidative stress and DNA damage in cancer cells. This secondary electron emission is crucial in achieving enhanced radiation efficacy, especially in radioresistant tumors that are difficult to eradicate using conventional radiotherapy alone. By concentrating these effects within the tumor microenvironment, Bi_2_O_3_ nanoparticles improve the therapeutic ratio—the balance between tumor control and normal tissue complication.^55,56^

Beyond their intrinsic radiosensitizing capabilities, Bi_2_O_3_ nanoparticles also exhibit versatile surface chemistry, enabling them to be functionalized with a wide range of ligands, polymers, and biomolecules. This tunable surface allows for improved dispersion in biological media, enhanced biocompatibility, and targeted delivery to tumor sites. For example, the conjugation of targeting ligands such as antibodies, peptides, or hyaluronic acid to Bi_2_O_3_ nanoparticles can facilitate active targeting of cancer cells that overexpress specific receptors. This enhances nanoparticle accumulation within tumors *via* receptor-mediated endocytosis, thereby increasing radiosensitization efficacy at the disease site while reducing systemic toxicity.^57–59^

In addition to radiosensitization, Bi_2_O_3_ nanoparticles show promise in cancer diagnosis and multimodal therapy, fulfilling the CDT paradigm. Their high electron density and atomic number make them suitable as contrast agents for X-ray-based imaging modalities, such as computed tomography (CT). This diagnostic capability allows clinicians to precisely localize tumors and monitor nanoparticle distribution in real-time, offering image-guided radiotherapy opportunities. Furthermore, the large surface area and modifiable surface chemistry of Bi_2_O_3_ nanoparticles make them ideal platforms for co-delivery of therapeutic agents, including chemotherapeutic drugs or photosensitizers, allowing for combined chemo-radiotherapy or photo-radiotherapy regimens.^41^

Preclinical studies have validated these capabilities. Research has shown that Bi_2_O_3_ nanoparticles enhance radiation-induced cytotoxicity in multiple cancer cell lines and animal models. Notably, the degree of radiosensitization is influenced by factors such as particle size, shape, and surface functionalization. Smaller Bi_2_O_3_ nanoparticles or those with rod-like morphologies have demonstrated superior cellular uptake and radiation enhancement effects due to increased surface-to-volume ratios and better interaction with tumor tissue. These findings underscore the importance of optimizing nanoparticle design to maximize clinical outcomes.^59,60^

**Fig. 4:**
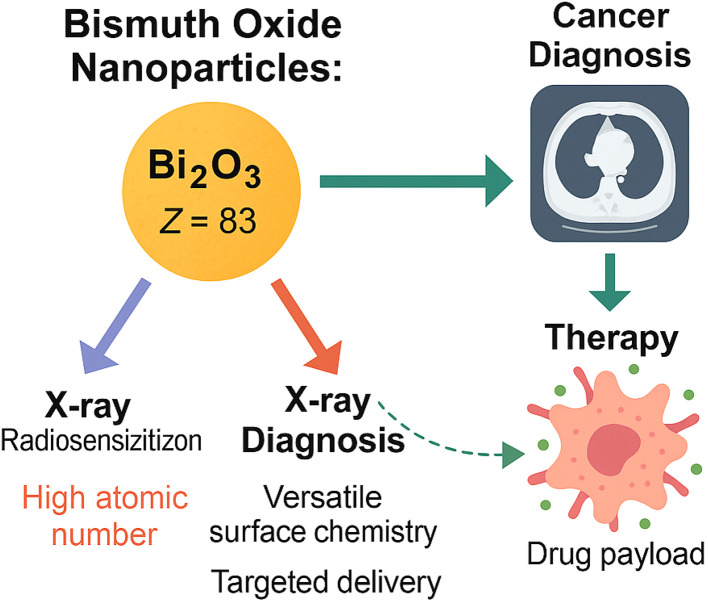
Schematic illustration of the radiosensitizing and chemodynamic therapy (CDT) potential of bismuth oxide (Bi_2_O_3_) nanoparticles.

Moreover, the relatively low inherent toxicity of bismuth compared to other high-*Z* elements like gold or platinum positions Bi_2_O_3_ as a safer alternative for *in vivo* applications. Studies have shown that properly engineered Bi_2_O_3_ nanoparticles exhibit favorable pharmacokinetics and biodistribution, with minimal accumulation in non-target organs and efficient clearance pathways.^60,61^ Nonetheless, surface modifications remain essential for mitigating any residual toxicity and for promoting colloidal stability in physiological environments. Bismuth oxide (Bi_2_O_3_) nanoparticles represent a cutting-edge solution for improving the precision and effectiveness of cancer radiotherapy. Their high atomic number and X-ray absorption capacity drive potent radiosensitization effects, while their adaptable surface chemistry supports targeted delivery, biocompatibility, and multifunctionality.^62^ These properties collectively position Bi_2_O_3_ nanoparticles as powerful agents within the framework of cancer diagnosis and therapy, offering promising pathways for more effective and less toxic treatment strategies in modern oncology.^63^ As illustrated in Fig. 4, their multifaceted roles in radiosensitization, targeted delivery, and diagnostic imaging underscore their integral value in the evolving landscape of precision oncology.

This potential was first rigorously examined by Stewart *et al.* (2016), who provided pioneering evidence that Bi_2_O_3_ nanoparticles can enhance radiosensitivity in highly resistant tumor cells.^36^ Their study, conducted on 9 L gliosarcoma cells, demonstrated significant radiosensitization under both kilovoltage (125 kVp) and megavoltage (10 MV) X-ray irradiation. Specifically, sensitization enhancement ratios (SERs) of 1.48 and 1.25 were observed, respectively, and these effects were attributed to the high-*Z* nature of bismuth, which enhances photoelectric absorption and secondary electron production, as well as to the unique platelet morphology of the nanoparticles. Monte Carlo simulations in this study further confirmed the interaction between X-rays and Bi_2_O_3_ nanoparticles, elucidating the mechanisms behind their ability to amplify radiation-induced damage in tumor cells.

Building upon this foundational work, Du *et al.* (2017) sought to overcome the limitations associated with tumor radioresistance and poor radiation targeting by developing hyaluronic acid-functionalized Bi_2_O_3_ nanoparticles (HA-Bi_2_O_3_ NPs).^32^ These nanoparticles were designed for active targeting of tumors overexpressing CD44 receptors and were synthesized *via* a one-pot hydrothermal method. The resulting HA-Bi_2_O_3_ NPs showed excellent water solubility, biocompatibility, and selective cellular uptake by cancer cells. *In vitro* studies demonstrated that these functionalized NPs not only significantly enhanced radiosensitivity in a dose-dependent manner but also effectively arrested the cell cycle and induced apoptosis when used alongside X-ray radiation. Moreover, their high X-ray attenuation properties enabled precise CT imaging, making them dual-functional theranostic agents. The study demonstrated that these surface-modified nanoparticles could serve as both contrast agents and radiosensitizers, providing a highly localized and efficient radiotherapeutic approach.

The therapeutic efficiency of Bi_2_O_3_ nanoparticles, however, is not solely dependent on their elemental composition or surface modification but also significantly influenced by their physical dimensions. Jamil *et al.* (2021) explored the effect of nanoparticle size on radiosensitization using Bi_2_O_3_ nanorods (NRs) across different megavoltage photon and electron beam therapies.^64^ MCF-7 and HeLa cancer cell lines were treated with Bi_2_O_3_-NRs ranging from 60 nm to 90 nm in size, and the results showed that the smallest nanorods (60 nm) yielded the highest SER, particularly in photon beam therapy. This enhanced radiosensitization effect was attributed to better cellular uptake and increased surface area, which promote greater interaction with ionizing radiation. Interestingly, MCF-7 cells exhibited higher radiosensitivity compared to HeLa cells, indicating cell-specific responses to nanoparticle-mediated sensitization. Notably, photon beam therapy produced a more significant SER than electron beam therapy, likely due to the higher energy transfer and increased probability of Compton scattering events that enhance ROS production in the presence of high-*Z* materials like Bi.

The importance of ROS generation in mediating the radiosensitizing effects of Bi_2_O_3_ nanoparticles was further elucidated by Sisin *et al.* (2020), who investigated the synergistic application of BiONPs with cisplatin (Cis) and baicalein-rich fraction (BRF), a plant-derived antioxidant compound.^65^ The study analyzed the combined effects of these agents under various clinical radiation modalities including high-dose-rate (HDR) brachytherapy, photon beam, and electron beam irradiation. Results showed that while Cis independently produced the highest levels of ROS, combinations involving BiONPs—especially BiONPs-Cis (BC)—further amplified ROS production, particularly under photon beam exposure. This ROS overproduction significantly increased DNA damage and apoptosis in cancer cells while sparing normal fibroblasts, suggesting selective cytotoxicity. Among the tested combinations, BC showed the highest SER in MCF-7 cells, reaffirming the synergistic potential of combining BiONPs with conventional chemotherapeutic agents to potentiate radiotherapy. Furthermore, brachytherapy appeared to benefit most from nanoparticle-assisted radiosensitization, perhaps due to its more localized and intense radiation dose delivery, further supporting the versatility of Bi_2_O_3_ nanoparticles across radiation types.

Despite these promising findings, challenges still persist in translating Bi_2_O_3_ nanoparticle-based radiosensitizers into clinical applications. Wen *et al.* (2022) reviewed recent advancements and obstacles in the use of bismuth-based nanoparticles for radiotherapy, emphasizing the need for precise tumor targeting, improved biocompatibility, and rapid clearance to minimize systemic toxicity.^33^ The review highlighted that simple bismuth nanoparticles may fall short in clinical efficacy due to limited tumor penetration, poor biodegradation, and immune resistance. However, surface modification, such as hyaluronic acid coating, and integration with other functional elements like iron ions, can significantly enhance diagnostic and therapeutic precision. Additionally, optimizing the structural properties of Bi_2_O_3_ nanoparticles—such as size, shape, and surface area—can increase drug loading efficiency and improve radiosensitization. The multifunctional role of Bi-based NPs is further strengthened when they are combined with other therapies like photothermal therapy (PTT) or high-intensity focused ultrasound (HIFU), offering a platform for synergistic and multimodal cancer treatment.

In a nut shell, from the initial validation of Bi_2_O_3_ nanoparticles as radiosensitizers by Stewart *et al.* (2016),^36^ through their structural optimization and functionalization as demonstrated by Du *et al.* (2017)^32^ and Jamil *et al.* (2021),^64^ to the demonstration of their synergistic behavior with chemotherapeutics by Sisin *et al.* (2020), and culminating in a comprehensive perspective on their design and clinical promise by Wen *et al.* (2022), a coherent and advancing narrative emerges.^33^ Bi_2_O_3_ nanoparticles clearly possess the potential not only to amplify radiotherapeutic outcomes through radiosensitization and enhanced imaging but also to serve as foundational elements in next generation theranostic platforms, especially when combined with complementary therapies and thoughtful design modifications to overcome current clinical limitations.

#### 3.1.2 Bismuth sulfide (Bi_2_S_3_) nanorods: dual photothermal and chemodynamic properties

Bismuth sulfide (Bi_2_S_3_) nanorods exhibit significant dual photothermal and chemodynamic therapeutic properties, making them promising candidates for multimodal cancer therapy. Their photothermal property arises from strong near-infrared (NIR) absorption, particularly around 808 nm, which allows them to efficiently convert absorbed light into localized heat. This photothermal effect can induce hyperthermia in tumor tissues, leading to cancer cell apoptosis with minimal damage to surrounding healthy tissues. The rod-like morphology and crystalline structure of Bi_2_S_3_ nanorods enhance their light-harvesting ability and improve photothermal conversion efficiency compared to spherical or larger-sized counterparts.^66–77^

In addition to photothermal therapy (PTT), Bi_2_S_3_ nanorods also exhibit chemodynamic therapy (CDT) potential. This is primarily attributed to their ability to catalyze Fenton-like reactions, especially when doped with or combined with transition metal ions such as Fe^2+^ or Mn^2+^. In the tumor microenvironment, which is rich in hydrogen peroxide (H_2_O_2_) and slightly acidic, these nanorods can facilitate the decomposition of H_2_O_2_ into highly toxic hydroxyl radicals (˙OH). These reactive oxygen species induce oxidative stress and damage cellular components, thereby enhancing cancer cell death through CDT.

Furthermore, Bi_2_S_3_ nanorods are often engineered into composite structures or functionalized with other therapeutic agents to improve their performance. For example, when integrated into core–shell structures with metal–organic frameworks (*e.g.*, ZIF-8), or conjugated with chemotherapeutic drugs, the resulting multifunctional nanoplatforms offer synergistic effects through the combination of PTT, CDT, and chemotherapy. Their strong X-ray attenuation properties also make them suitable for computed tomography (CT) imaging, allowing for image-guided therapy.^69^

For example, in the study by Liu *et al.* (2015), bismuth sulfide (Bi_2_S_3_) nanorods (NRs) are presented as a multifunctional nanoplatform that simultaneously enables dual photothermal and chemodynamic properties, making them a potent candidate for cancer theranostics.^70^ These nanorods exhibit strong near-infrared (NIR) absorption, which underpins their excellent photothermal conversion efficiency and makes them suitable for multispectral optoacoustic tomography (MSOT) and X-ray computed tomography (CT) imaging. This dual-modality imaging capability not only aids in high-resolution imaging but also in guiding and monitoring the therapeutic process, ensuring accurate and minimally invasive tumor ablation.

**Fig. 5:**
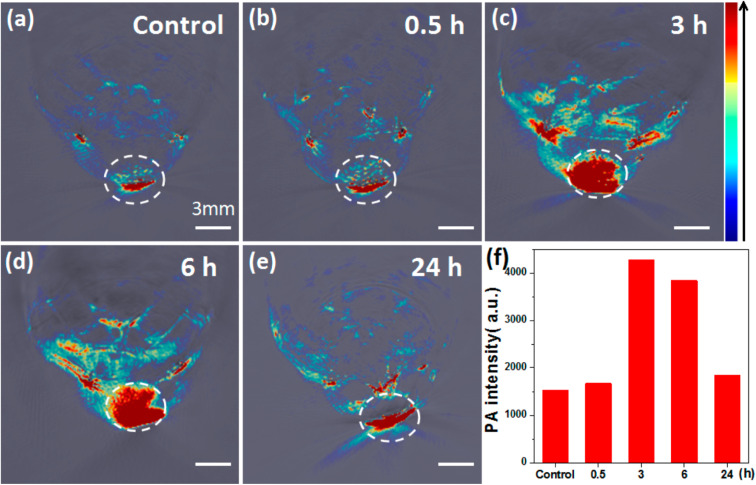
*In vivo* multispectral optoacoustic tomography (MSOT) imaging of 4T1 tumor-bearing mice following intravenous administration of Bi_2_S_3_ nanorods. (a–e) Representative MSOT cross-sectional images of tumors acquired at baseline (pre-injection) and at 0.5, 3, 6, and 24 hours post-intravenous injection of Bi_2_S_3_ NRs, highlighting the progressive accumulation and enhanced contrast in the tumor region. (f) Quantitative analysis of photoacoustic (PA) signal intensity within the tumor over time, showing a significant increase peaking at 3 hours post-injection and sustained signal up to 24 hours, indicating effective tumor homing and retention of the nanorods.^70^

The MSOT capability of Bi_2_S_3_ NRs was investigated by intravenously injecting tumor-bearing mice with 200 μL of Bi_2_S_3_ NRs (2 mg mL^−1^). The photoacoustic (PA) signals in tumors were observed to increase significantly over time, suggesting the gradual accumulation and retention of the nanorods in tumor tissues. Notably, prior to injection, the tumors showed only weak PA signals due to blood contrast (∼1500 a.u.). However, 3 hours post-injection, a sharp increase in PA signal intensity (∼4270 a.u.) was observed, which persisted up to 24 hours. This clearly demonstrated the efficient passive targeting of Bi_2_S_3_ NRs to tumor sites due to their optimal size and surface characteristics, likely enhancing the enhanced permeability and retention (EPR) effect (Fig. 5a–f).

**Fig. 6:**
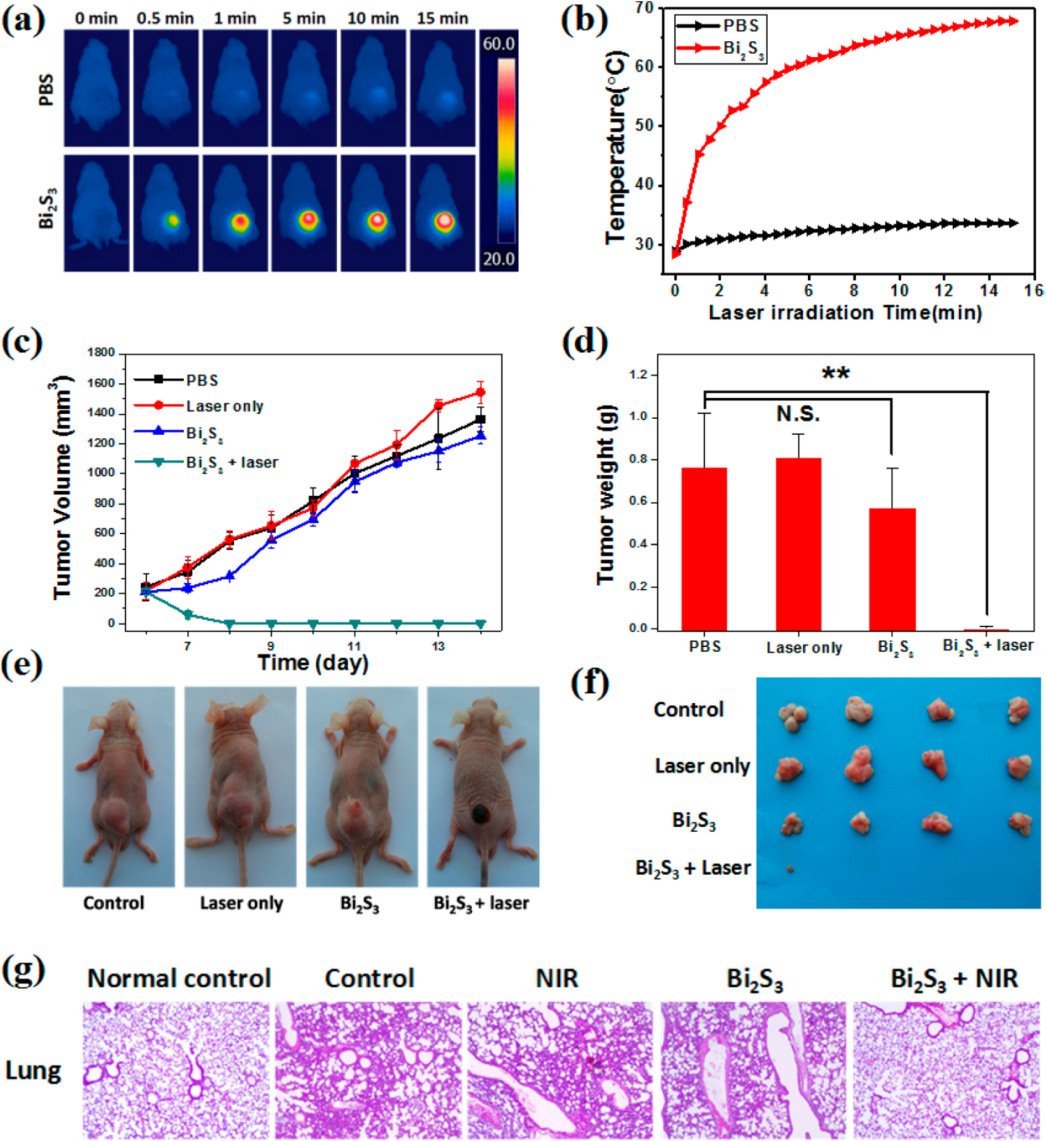
*In vivo* photothermal therapy evaluation using Bi_2_S_3_ nanorods. (a and b) Near-infrared (NIR) thermal images of 4T1 tumor-bearing mice intravenously injected with PBS or Bi_2_S_3_ NRs (dose = 20 mg kg^−1^), followed by 808 nm laser irradiation at a power density of 1 W cm^−2^, captured at various time intervals. (c) Tumor volume growth curves of different treatment groups over time (*n* = 4 per group), including: (a) PBS only, (b) laser only, (c) Bi_2_S_3_ NRs only, and (d) Bi_2_S_3_ NRs + laser. (d) Average tumor weights harvested at the end of the treatment period, with statistical analysis performed using a two-tailed *t*-test (*p* < 0.01; N.S., not significant, *p* > 0.05). (e) Representative images of mice from each treatment group taken 14 days post-therapy. (f) Photographs of excised tumors from all groups at the conclusion of the treatment period. (g) Hematoxylin and eosin (H&E) staining of tumor tissues show no significant metastasis in the Bi_2_S_3_ NRs + laser group, confirming the therapeutic efficacy and suppression of tumor spread. Error bars represent mean ± standard deviation.^70^

Additionally, the Bi_2_S_3_ NRs served as effective CT contrast agents, attributed to the high atomic number of bismuth, which affords superior X-ray attenuation compared to traditional iodine-based agents. *In vitro* studies demonstrated a linear increase in Hounsfield Units (HU) with rising Bi_2_S_3_ NR concentrations, surpassing that of clinical iopromide. More importantly, *in vivo* CT imaging further confirmed this contrast enhancement. At 3 hours post-injection, the tumor sites exhibited marked CT contrast, consistent with the MSOT data and affirming the accumulation and retention of Bi_2_S_3_ NRs in the tumor microenvironment. This correlation was critical in establishing the real-time monitoring capacity of these nanorods for both imaging and therapy (Fig. 6).

The dual photothermal and chemodynamic functionalities of Bi_2_S_3_ NRs thus offer significant advantages: photothermal therapy (PTT) benefits from their efficient NIR-induced heat generation, while their capacity to enable high-resolution, non-invasive imaging *via* MSOT and CT enhances diagnostic precision and therapeutic monitoring. Their passive tumor-targeting ability, long retention time, and biocompatibility further support their role as a “satellite” for imaging and a “precision weapon” for therapy, highlighting their promise in the field of precision cancer nanomedicine.

Jiang *et al.* (2020) developed defect-rich bismuth sulfide (Bi_2_S_3_) nanorods with dual functionality for both photothermal therapy (PTT) and chemodynamic therapy (CDT), addressing the historically poor photothermal efficiency of Bi_2_S_3_-based systems.^66^ By tailoring the morphology and introducing structural defects, they achieved a strong near-infrared (NIR) absorption band, enabling a remarkable photothermal conversion efficiency (PCE) of 78.1%. This high PCE, significantly outperforming earlier reported semiconductor photothermal agents, was attributed to the metal-like absorption behavior arising from the defect structure, and a blue-shifted absorption peak due to the nanorods' unique geometry. Experimental validation demonstrated that under 808 nm laser irradiation, the Bi_2_S_3_ nanorods effectively elevated temperatures sufficient for cancer cell ablation both *in vitro* and *in vivo*.

The dual functionality of the nanorods extended beyond PTT. Leveraging the high atomic number of bismuth, the Bi_2_S_3_ nanorods also served as potent contrast agents for computed tomography (CT) imaging. CT signal intensity was shown to increase linearly with nanorod concentration, with a high HU value slope of 27.1 HU L g^−1^, indicating superior imaging capability compared to other reported nanomaterials. *In vivo*, following intratumoral injection, a significant enhancement in CT contrast was observed at the tumor site, with HU values increasing from 17 to 125, clearly delineating the tumor tissue.

**Fig. 7:**
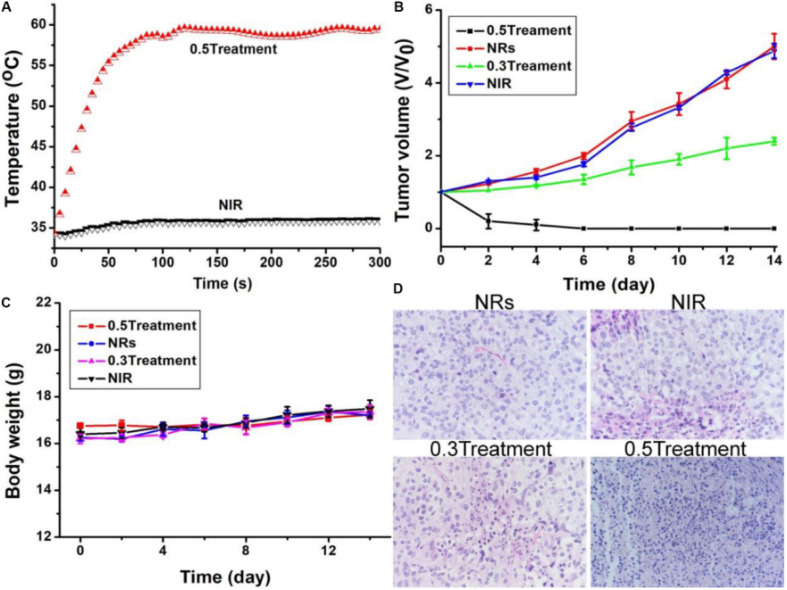
(A) *In vivo* photothermal response following injection of PBS or Bi_2_S_3_ nanorods, under 808 nm laser irradiation at a power density of 0.5 W cm^−2^. (B) Tumor volume progression and (C) body weight changes observed across different treatment groups. (D) Hematoxylin and eosin (H&E) stained histological images of excised tumor sections from each group, captured at 200× magnification.^66^

To assess *in vivo* photothermal efficacy, tumor-bearing mice were divided into four groups: PBS only, laser only, Bi_2_S_3_ nanorods only, and Bi_2_S_3_ nanorods combined with laser irradiation. Thermal imaging confirmed that mice receiving both nanorods and laser treatment showed a rapid increase in surface tumor temperature to ∼55 °C within 100 seconds, whereas minimal temperature change was observed in control groups (Fig. 7A). This result demonstrated the potent *in vivo* photothermal response of the Bi_2_S_3_ nanorods.

Following treatment, tumor volume was monitored over time. Mice in the Bi_2_S_3_ + laser group showed complete tumor regression with no recurrence, whereas the Bi_2_S_3_-only group exhibited partial suppression and the other controls showed unimpeded tumor growth (Fig. 7B). These findings confirm that both nanorods and laser irradiation are required for full therapeutic effect. Notably, the treatment exhibited no observable systemic toxicity, as evidenced by consistent body weights across all groups (Fig. 7C).

Histopathological analysis using hematoxylin and eosin (H&E) staining further supported the therapeutic efficacy. Tumors treated with the combined Bi_2_S_3_ and laser approach revealed extensive tissue damage, characterized by disrupted morphology and nuclear condensation, whereas untreated or singly treated groups retained intact tissue architecture (Fig. 7D). These results affirm that Bi_2_S_3_ nanorods act not only as highly efficient photothermal agents but also hold potential for integration with CT imaging for image-guided cancer therapy. The dual photothermal and diagnostic properties position Bi_2_S_3_ nanorods as a promising theranostic platform in oncology.

Yang *et al.* (2019) addressed a significant limitation in combinatorial phototherapies namely, the dependence on multi-component systems or dual-laser activation by synthesizing ultrasmall, water-dispersible polylysine-coated Bi_2_S_3_ quantum dots (PLL-Bi_2_S_3_ QDs) *via* a high-temperature organic method and ligand exchange strategy.^71^ These QDs not only exhibited strong near-infrared (NIR) absorption at 808 nm but also demonstrated impressive photothermal conversion efficiency and rapid generation of reactive oxygen species (ROS), attributed to their ultrasmall size and sulfide-enriched surfaces. This synergy between photothermal therapy (PTT) and photodynamic therapy (PDT) within a single nanostructure enabled more effective cancer cell ablation than traditional larger Bi_2_S_3_ nanorods, as confirmed through *in vitro* and *in vivo* studies. Additionally, their excellent biocompatibility further supports their potential clinical application as single-laser-activated therapeutic agents.

Complementing this, Cheng *et al.* (2018) explored the photothermal potential of Bi_2_S_3_ nanorods by investigating their intrinsic deep-level defects (DLDs), which act as electron–hole recombination centers that enhance phonon generation and, consequently, photothermal efficiency.^72^ To further amplify this property, Bi_2_S_3_–Au heterojunction nanorods were engineered, leveraging the synergistic effect of gold to intensify DLD activity. Under 808 nm laser irradiation, these heterojunction nanorods induced heightened cellular responses, such as increased expression of heat shock protein 70 and a higher rate of apoptosis, ultimately resulting in more pronounced tumor suppression. These findings emphasized the critical role of defect engineering and nanostructure design in optimizing the photothermal properties of Bi_2_S_3_-based materials for effective cancer therapy, especially when integrated with imaging modalities like computed tomography.

Building on the multifunctionality of Bi_2_S_3_ nanorods, Dash *et al.* (2024) developed an advanced core–shell nanostructure by integrating Bi_2_S_3_ nanorods with a Fe/Mn-doped zeolitic imidazolate framework (ZIF-8), forming Bi_2_S_3_@FMZ (B@FMZ) nanoparticles.^53^ This design not only capitalized on the photothermal efficacy of Bi_2_S_3_ under 808 nm laser irradiation but also introduced chemodynamic therapy (CDT) capabilities *via* Fenton-like reactions catalyzed by Fe^2+^/Mn^2+^ ions. These ions efficiently decomposed H_2_O_2_ in the tumor microenvironment, generating cytotoxic hydroxyl radicals that contributed to enhanced cancer cell apoptosis. Moreover, the porous ZIF-8 structure enabled high-capacity loading of methotrexate (MTX), and the photothermal effect facilitated controlled drug release, achieving up to 87.25% release at acidic pH under NIR irradiation. The B@FMZ/MTX nanoparticles demonstrated substantial antitumor efficacy in both *in vitro* and *in vivo* studies, driven by the integrated effects of PTT, CDT, and chemotherapy, while maintaining good biocompatibility. Additionally, these nanoparticles were suitable for dual-modal imaging, offering guidance through both magnetic resonance and computed tomography techniques.

Together, these studies exemplify the versatile therapeutic and diagnostic potential of Bi_2_S_3_ nanorods, particularly when engineered into multifunctional platforms. The ability to combine photothermal heating with ROS generation and Fenton-like reactions within a single nanostructure opens new pathways for efficient, targeted cancer treatments. These findings underscore the importance of nanoscale design, surface engineering, and component integration in developing next-generation nanomedicines.

#### 3.1.3 Bismuth vanadate (BiVO_4_): catalytically active for CDT and photoresponsive

Bismuth vanadate (BiVO_4_) has emerged as a highly promising nanomaterial for cancer treatment due to its unique photocatalytic and chemodynamic properties. Structurally, BiVO_4_ possesses a monoclinic scheelite crystal phase with a narrow bandgap (∼2.4 eV), enabling strong absorption in the visible-light region. This photoresponsive behaviour makes it particularly suitable for light-driven therapeutic applications, including photodynamic therapy (PDT) and photo-enhanced chemodynamic therapy (CDT).^72,73^

**Fig. 8:**
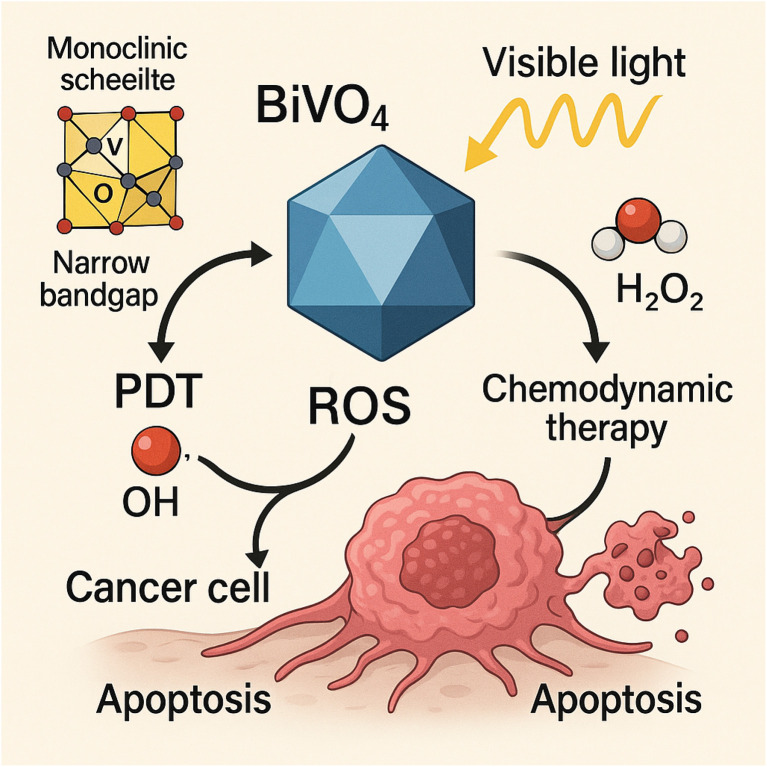
The multifunctional role of bismuth vanadate (BiVO_4_) in cancer therapy.

In the context of CDT, BiVO_4_ exhibits notable catalytic activity in generating reactive oxygen species (ROS), especially hydroxyl radicals (˙OH), *via* Fenton-like or photo-Fenton-like reactions. In the tumor microenvironment, BiVO_4_ can accelerate the decomposition of hydrogen peroxide (H_2_O_2_), which is typically overexpressed in cancer cells, into toxic ·OH radicals. These ROS species inflict oxidative damage on cancer cells, leading to their selective apoptosis while sparing healthy tissues. Furthermore, when activated by visible or near-infrared (NIR) light, BiVO_4_ generates electron–hole pairs that further enhance ROS production through photocatalysis, reinforcing the CDT effect in a synergistic manner (Fig. 8).^74–76^

Moreover, BiVO_4_ can be integrated with other therapeutic agents or nanostructures to improve its tumor selectivity, bioavailability, and ROS yield. Surface functionalization with biocompatible polymers or targeting ligands enhances its dispersibility and active accumulation in tumor tissues, while also offering platforms for multimodal therapy. Some studies have demonstrated that coupling BiVO_4_ with metal ions such as Fe^3+^ or Mn^2+^ can amplify its catalytic activity and broaden its therapeutic window.^72,73^ Importantly, BiVO_4_'s photoactivation ability not only supports enhanced ROS generation but also allows for real-time imaging and therapy guidance when coupled with suitable imaging modalities. The dual advantage of being both catalytically active and photoresponsive positions BiVO_4_ as a multifunctional theranostic agent for precision oncology. Bismuth Vanadate stands out as a photoresponsive nanomaterial with robust catalytic properties for CDT. Its ability to harness both endogenous and exogenous stimuli (H_2_O_2_ and light, respectively) for ROS generation makes it a compelling candidate for synergistic cancer therapy strategies. Future research focused on optimizing its biocompatibility, targeting capabilities, and integration with multimodal platforms could further enhance its clinical translation potential.^72–74^

Recent research has positioned bismuth vanadate (BiVO_4_) as a promising photocatalytic and chemodynamic agent for cancer therapy due to its visible light activity, ROS-generating capabilities, and biocompatibility. However, while BiVO_4_ demonstrates strong potential in phototherapy, its clinical translation faces several challenges, including inefficient electron–hole separation and tumor hypoxia—issues that severely hinder therapeutic outcomes in photodynamic (PDT) and sonodynamic therapy (SDT).^77,78^

In addressing these limitations, Nagubandi *et al.* (2024) developed a hybrid Ti_3_C_2_–BiVO_4_ nanosheet composite, integrating the photocatalytic activity of BiVO_4_ with the high conductivity and surface area of MXene Ti_3_C_2_.^77^ The composite was synthesized *via* hydrothermal treatment and characterized by techniques such as XRD, FESEM, and EDS, confirming successful heterostructure formation. The hybrid structure significantly enhanced charge separation under visible light, boosting the generation of ROS such as hydroxyl and superoxide radicals. These ROS caused mitochondrial damage, lipid peroxidation, and DNA fragmentation in colorectal cancer (CRC) cells, culminating in apoptosis. Furthermore, the Ti_3_C_2_–BiVO_4_ nanosheets promoted cell cycle arrest and suppressed cancer progression through the downregulation of PI3K/AKT/mTOR, MAPK/ERK, and Wnt/β-catenin pathways. These results point to the composite's utility in photo-enhanced chemodynamic therapy (CDT) with additional immunomodulatory and antiproliferative benefits.

A complementary approach is provided by Yang *et al.* (2022), who tackled the limitations of sonodynamic therapy (SDT)—notably, the poor electron–hole separation efficiency of traditional sonosensitizers and the hypoxic nature of the tumor microenvironment.^78^ In their study, they fabricated photoetched BiVO_4_ nanorods (PEBVO@PEG NRs) functionalized with DSPE-PEG2000. The photoetching process introduced surface oxygen vacancies, which are known to act as active sites for charge separation, significantly improving the ROS generation efficiency under ultrasound (US) stimulation. Importantly, these nanorods exhibited self-sufficient oxygen generation, directly addressing tumor hypoxia, a critical barrier in many tumor treatment modalities.

The PEBVO@PEG nanorods demonstrated enhanced SDT effects in hypoxic tumors by coupling oxygen self-supply with elevated ROS generation—mechanistically improving upon traditional sonosensitizers. The DSPE-PEG2000 coating imparted stability and biocompatibility, promoting tumor accumulation and prolonging systemic circulation. This innovation not only augmented the therapeutic index of SDT but also revealed new potential for BiVO_4_-based nanomaterials in non-invasive, deeply penetrating, and controllable sonodynamic treatments.

Together, these three studies highlight a growing trend in BiVO_4_ nanomedicine: the strategic modification of BiVO_4_'s structure or surface to overcome intrinsic limitations in ROS generation and microenvironmental challenges. Whether through composite engineering with conductive nanomaterials (as in Ti_3_C_2_–BiVO_4_) or photoetching to introduce oxygen vacancies (as in PEBVO@PEG), the enhancements aim to increase charge separation efficiency and bolster ROS/O_2_ production. Both approaches successfully translated these physicochemical improvements into biological outcomes, achieving superior cancer cell apoptosis under controlled activation.

Moreover, these advances showcase the adaptability of BiVO_4_ for different therapy modalities: photo-enhanced CDT under visible light and US-activated SDT under deep-penetrating ultrasound. This dual functionality paves the way for combination or multimodal therapy, leveraging the strengths of both light- and ultrasound-responsive systems to broaden the therapeutic window and address tumor heterogeneity.

The works of Nagubandi *et al.* (2024)^77^ and Yang *et al.* (2022)^78^ collectively validate the potential of engineered BiVO_4_ nanostructures in overcoming the longstanding hurdles of poor electron–hole separation and tumor hypoxia. These studies set a foundation for future innovations in ROS-amplifying, oxygen-generating nanoplatforms, aimed at improving the precision, depth, and efficacy of cancer nanotherapy.

### 3.2 Surface-engineered bismuth-based nanoparticles for targeted cancer therapy

Bismuth-based nanoparticles have emerged as versatile and powerful tools for targeted cancer therapy, owing to their unique physicochemical attributes such as high X-ray attenuation, favorable biocompatibility, and facile functionalization potential. Recent advances in nanomedicine have increasingly focused on surface engineering strategies to optimize their biological performance, targeting ability, and therapeutic efficacy. By modifying the surface of bismuth nanoparticles with various biocompatible and functional moieties, researchers are enhancing their solubility, stability, biodistribution, and tumor-specific accumulation while minimizing off-target toxicity.^79,80^

For instance, Alex and Mathew (2023) demonstrated an innovative surface modification strategy for bismuth oxide (Bi_2_O_3_) nanoparticles using functionalized beta-cyclodextrin (β-CD) as a biocompatible carrier.^79^ The Bi_2_O_3_ nanoparticles were synthesized using polyvinyl alcohol (PVA) as a reductant, and β-CD was functionalized with biotin through Steglich esterification, enabling effective bio-conjugation. The resulting surface-modified Bi_2_O_3_ NPs, with particle sizes ranging from 12 to 16 nm, exhibited significant antibacterial and anticancer effects. Comprehensive characterization using FTIR, TEM, SEM, XRD, and DSC confirmed the successful fabrication and functionalization of the nanostructures. This work emphasizes the importance of engineering the nanoparticle surface with biologically active and target-specific ligands to improve their efficacy as therapeutic agents.

In another innovative approach, Zhao *et al.* (2018) addressed the challenges of poor tumor accumulation and rapid clearance associated with traditional nanodrug delivery systems by developing macrophage membrane (M)-camouflaged, quercetin-loaded hollow bismuth selenide nanoparticles (M@BS-QE NPs).^80^ This biomimetic surface engineering endowed the nanoparticles with immune evasion capabilities, extended circulation time, and enhanced tumor homing, primarily due to the presence of C–C chemokine ligand 2 (CCL2)-mediated recruitment and active targeting mechanisms. Under near-infrared (NIR) irradiation, quercetin was released to sensitize cancer cells to photothermal therapy (PTT) by downregulating heat shock protein 70 (HSP70), a protein that imparts thermoresistance to cancer cells. Simultaneously, the nanoplatform suppressed breast cancer metastasis by inhibiting the *p*-Akt/MMP-9 axis, showcasing its potential for both primary tumor ablation and metastasis inhibition. This study illustrates the power of surface bio-camouflage and multi-stimuli-responsive release systems in overcoming biological barriers and improving cancer therapeutic outcomes.

Surface engineering also plays a crucial role in enhancing the sonodynamic therapy (SDT) potential of bismuth-based systems. Yang *et al.* (2022) fabricated photoetched BiVO_4_ nanorods (PEBVO@PEG NRs) modified with DSPE-PEG_2000_ to tackle the limitations of electron–hole separation inefficiency and tumor hypoxia in conventional SDT.^78^ The photoetching process introduced abundant oxygen vacancies on the BiVO_4_ surface, thereby enhancing charge separation and increasing the generation of reactive oxygen species (ROS) and oxygen under ultrasound stimulation. This oxygen self-supplying capability significantly improved SDT efficacy in hypoxic tumor environments, marking a significant step toward the clinical translation of responsive nanotherapeutics.

Collectively, these studies underscore the critical role of surface modification in tailoring bismuth-based nanoparticles for targeted cancer therapy. Whether through chemical conjugation with biocompatible molecules like β-CD, biomimetic camouflaging with cell membranes, or structural tuning for ROS generation, surface-engineered nanoparticles offer multifunctional platforms that integrate imaging, targeted delivery, and effective tumor eradication. As these strategies evolve, future developments are expected to focus on smart, stimuli-responsive designs that further enhance selectivity, minimize systemic toxicity, and maximize therapeutic success in diverse cancer types.

### 3.3 Multifunctional hybrid systems

#### 3.3.1 BiNPs combined with MRI/CT contrast agents

Bismuth-based nanoparticles (BiNPs) have gained substantial attention as dual-modal imaging contrast agents, particularly in the integration of magnetic resonance imaging (MRI) and computed tomography (CT), due to their high atomic number, favorable biocompatibility, and unique physicochemical properties. The high atomic number of bismuth (*Z* = 83) imparts a strong X-ray attenuation coefficient, which makes it highly effective for enhancing contrast in CT imaging, often outperforming traditional iodine-based agents.^81,82^ At the same time, BiNPs can be engineered to possess or integrate with magnetic components, enabling their application in MRI, particularly for T_2_-weighted contrast. Their surface chemistry can be readily modified to allow conjugation with polymers, targeting ligands, or other functional moieties, improving their circulation time, biodistribution, and selective accumulation at disease sites.^83^

Additionally, the relatively low toxicity and good biocompatibility of bismuth compared to other heavy metals (*e.g.*, lead or cadmium) make BiNPs more suitable for biomedical applications. Their physicochemical versatility—such as tunable size, shape, surface charge, and payload capacity—further supports their development as multifunctional platforms not only for imaging but also for therapy (theranostics), where diagnostic and therapeutic capabilities are combined in a single system.^83,84^

The integration of bismuth-based nanoparticles (BiNPs) with MRI/CT imaging agents has led to promising advances in cancer theranostics, exemplified by the comprehensive study conducted by Li *et al.* (2018). In their work, a novel multifunctional theranostic nanoplatform—Tam-Bi_2_S_3_@mPS—was engineered to address prevailing limitations such as inefficient tumor accumulation and complex modification procedures.^85^ This core–shell nanocomposite consisted of rod-like Bi_2_S_3_ nanoparticles (a high atomic number material known for strong X-ray attenuation), encapsulated in mesoporous silica (mPS), loaded with doxorubicin (DOX), and surface-conjugated with trastuzumab (Tam), a monoclonal antibody targeting HER2-positive breast cancer. This construct provided a single-agent system capable of both precise imaging and effective therapeutic performance.

**Fig. 9:**
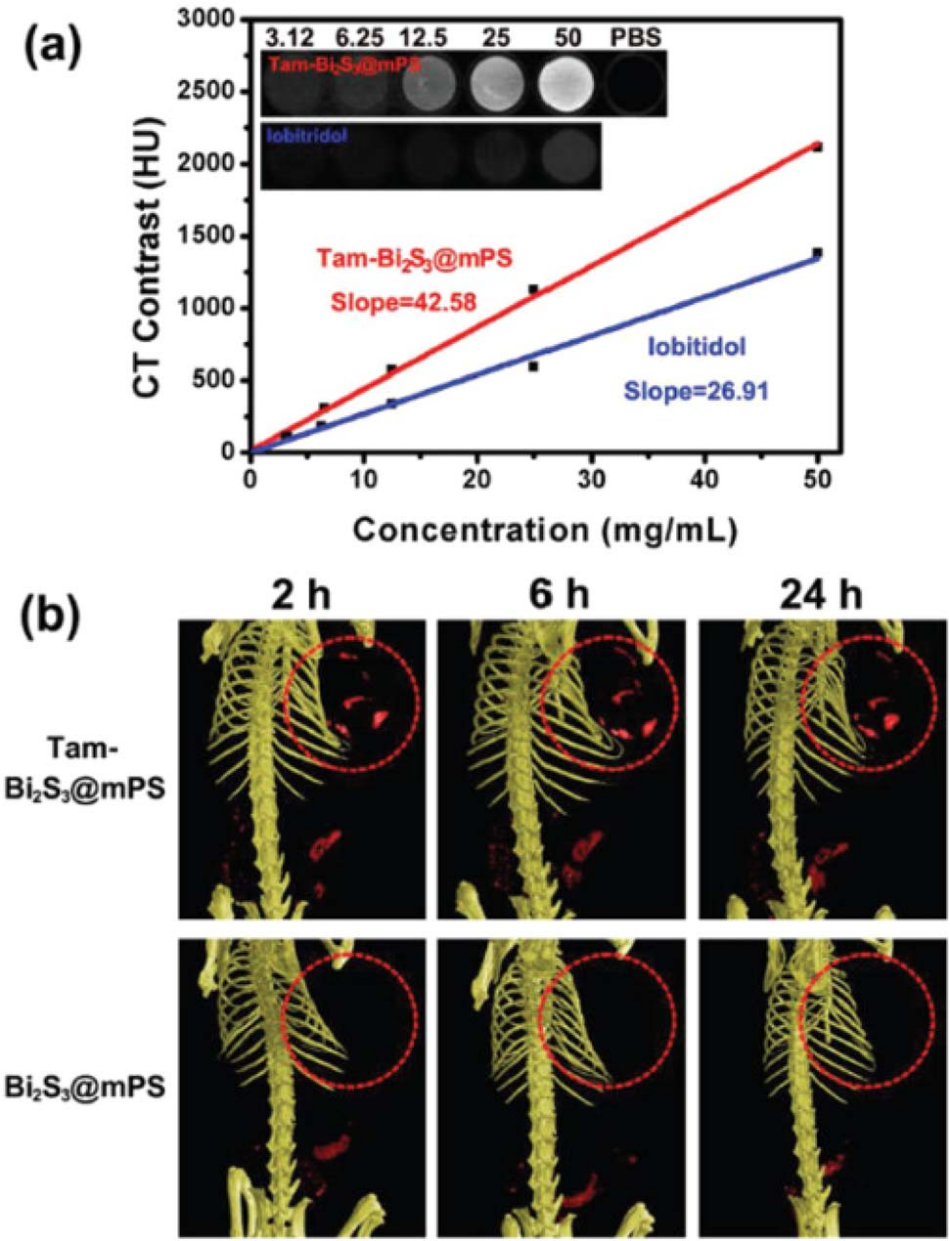
Enhanced *in vitro* and *in vivo* CT Imaging using Tam-Bi_2_S_3_@mPS nanoparticles. (a) CT attenuation values (HU) of Tam-Bi_2_S_3_@mPS and iobitridol at varying concentrations under 80 kV X-ray voltage. *Inset:* Representative *in vitro* CT images of Tam-Bi_2_S_3_@mPS and iobitridol suspensions at corresponding concentrations. (b) *In vivo* CT images of SKBR-3 tumor-bearing nude mice captured at 2, 6, and 24 hours post-intravenous injection of 200 μL of either Tam-Bi_2_S_3_@mPS or Bi_2_S_3_@PS (10 mg mL^−1^). Tumor regions are marked with red circles. These results demonstrate that Tam functionalization significantly improves nanoparticle accumulation at the tumor site, enabling enhanced CT imaging and tumor visualization.^85^

As a CT contrast agent, the Tam-Bi_2_S_3_@mPS nanoparticles exhibited remarkable imaging capabilities due to the inherent radiopacity of bismuth. Compared to iobitridol—a clinically approved third-generation CT contrast agent—the Tam-Bi_2_S_3_@mPS showed superior Hounsfield unit (HU) values at equivalent concentrations, with a contrast efficiency of 42.58 HU mL mg^−1^, outperforming iobitridol (26.91 HU mL mg^−1^), iopromide (16.38 HU mL mg^−1^), and even gold nanorods (31.26 HU mL mg^−1^). This enhanced contrast efficiency was clearly demonstrated in Fig. 9a, where the CT signal intensity increased linearly with concentration, confirming the platform's potential for dose-efficient imaging. Furthermore, Fig. 9b illustrated the strong *in vivo* CT imaging capability of the Tam-Bi_2_S_3_@mPS nanoparticles post intravenous administration in SKBR-3 tumor-bearing nude mice. A significant contrast enhancement at the tumor site was visible up to 24 hours after injection, underscoring the formulation's tumor targeting efficiency and retention. Importantly, control groups lacking trastuzumab modification showed negligible tumor accumulation, and ICP-MS data confirmed up to 16-fold higher bismuth content in tumors for the targeted Tam-Bi_2_S_3_@mPS group *versus* the untargeted equivalent, further validating the active targeting mechanism and tumor-specific accumulation.

**Fig. 10:**
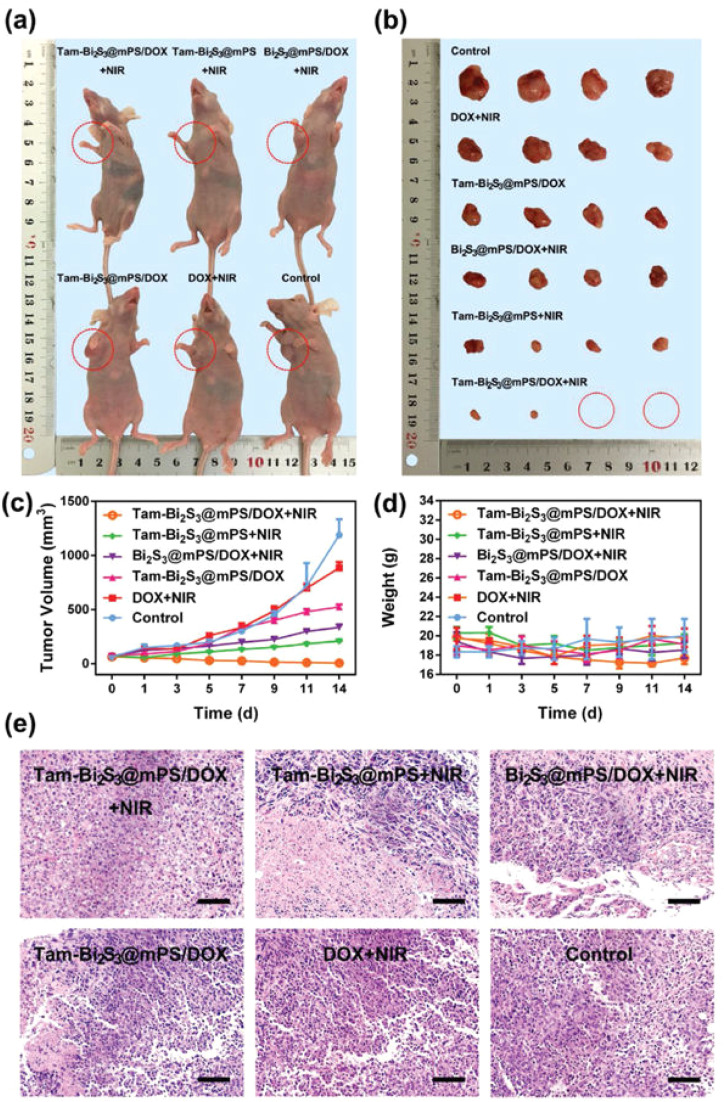
*In vivo* antitumor efficacy of various nanoparticle treatments, highlighting the superior synergistic effect of Tam-Bi_2_S_3_@mPS/DOX combined with NIR Irradiation. (a) Photographic images of SKBR-3 tumor-bearing nude mice from each treatment group, taken 14 days post-treatment. (b) Excised tumor specimens from the respective treatment groups after 14 days. (c) Tumor volume progression curves over 14 days, comparing therapeutic responses across groups. (d) Body weight changes of mice throughout the 14-day treatment period, indicating systemic toxicity or well-being. (e) H&E-stained tumor tissue sections from different groups, revealing histopathological changes post-treatment. Scale bar = 200 μm.^85^

Beyond imaging, the theranostic capability of the nanoparticles was evaluated for synergistic photothermal-chemotherapy. The inclusion of DOX enabled chemotherapy, while NIR laser irradiation leveraged the photothermal properties of Bi_2_S_3_ to enhance cytotoxic effects. *In vivo* therapeutic efficacy studies revealed that the combination of Tam-Bi_2_S_3_@mPS/DOX with NIR irradiation led to complete tumor ablation with no visible regrowth by day 14. This outcome is compellingly depicted in Fig. 10a and b, where treated mice and excised tumors from different groups are compared. Only the Tam-Bi_2_S_3_@mPS/DOX + NIR group showed significant tumor regression, while all other treatment arms, including those receiving monotherapies or untargeted NPs, exhibited partial or no tumor inhibition. Corresponding tumor volume progression data (Fig. 10c) reinforced this observation, showing the most profound and sustained tumor shrinkage in the targeted dual-modality group. Additionally, body weight monitoring (Fig. 10d) confirmed minimal systemic toxicity, and histological examination (Fig. 10e) demonstrated extensive tumor tissue damage only in the dual-treated Tam-Bi_2_S_3_@mPS/DOX + NIR group. This study by Li *et al.* offers a compelling demonstration of how Bi_2_S_3_-based nanoparticles, when integrated with both therapeutic and imaging modalities, can serve as highly effective platforms for cancer diagnosis and treatment. By uniting CT contrast enhancement, targeted delivery *via* HER2 recognition, photothermal activation, and chemotherapeutic drug release within a single nanostructure, the Tam-Bi_2_S_3_@mPS system exemplifies the future of image-guided precision oncology.

The study by Naha *et al.* (2014) offers a foundational insight into the synthesis of composite bismuth–iron oxide nanoparticles (BIONs), which effectively merge the strong X-ray attenuation of bismuth with the T2-weighted MR imaging capabilities of iron oxide.^86^ Utilizing a clinically approved dextran-coated iron oxide base, the researchers successfully synthesized a series of BIONs, optimizing the Bi-30 formulation for *in vivo* studies. This formulation demonstrated robust dual-modality performance, offering prolonged vascular contrast in CT and significant liver signal loss in MRI. Moreover, BIONs were shown to be biocompatible and biodegradable, with bismuth excretion primarily through the renal pathway. This underscores their potential for clinical translation by addressing both safety and diagnostic efficacy.

Complementing this approach, Hassan *et al.* (2019) explored dextrin-coated bismuth-doped manganese ferrite nanoparticles (Bi_0_._3_Mn_0_._55_Fe_2_O_4_) specifically designed for MRI contrast enhancement.^87^ These nanoparticles displayed superparamagnetic behavior and were optimized for use in T2-and T2*-weighted imaging sequences. The co-precipitation method yielded ultrasmall particles (∼4 nm) with high *r*_2_ and *r*_2_* relaxivity values and negligible cytotoxicity, making them particularly promising for tumor-targeted imaging. The enhanced contrast achieved in T2*-weighted imaging highlights the value of bismuth doping in magnetic nanostructures to further tune MRI responsiveness while preserving biosafety.

Further innovations were demonstrated by Goji *et al.* (2024), who developed a multifunctional nano-metal–organic framework (nanoMOF) composed of bismuth, zirconium, and porphyrin.^88^ This hybrid structure integrates diagnostic imaging with therapeutic functions, notably doxorubicin delivery, photodynamic therapy (PDT), and radiation therapy (RT). The PEGylated and aptamer-functionalized construct (Apt@DOX) not only enhanced biocompatibility and tumor targeting but also facilitated CT imaging, illustrating the utility of bismuth in high-contrast X-ray visualization. In both *in vitro* and *in vivo* settings, this platform showed synergistic tumor suppression when combined with PDT and RT, affirming the versatility of bismuth-containing systems for comprehensive cancer theranostics.

In contrast to nanoparticle-based systems, Fu et *al.* (2020) introduced a small molecule bismuth chelate using diethylenetriaminepentaacetic acid (DTPA) as a chelating ligand, serving as a straightforward and effective CT contrast agent.^89^ This chelates demonstrated superior renal CT imaging compared to the conventional iodinated agent iohexol, with markedly improved CT values and minimal toxicity up to 500 μM. Its pharmacokinetic profile—characterized by rapid clearance and a short half-life—positions it as a safe and efficient alternative to traditional CT agents. This work is pivotal in showcasing how simplified molecular design can meet clinical demands without compromising safety or efficacy.

Expanding the diagnostic utility further, Peng *et al.* (2025) developed BiNPs integrated with fluorescent dyes (FITC and ICG) for bimodal CT and fluorescence imaging.^90^ These bismuth-loaded poly(α-amino acid) nanoparticles not only provided excellent X-ray attenuation but also sustained fluorescence emissions, enabling deep tissue imaging in CT and sensitive cellular visualization through fluorescence. Notably, their catechol-rich polymer shell conferred antioxidant properties, potentially mitigating inflammatory responses during *in vivo* applications. The prolonged imaging signal and dual-modality functionality exemplify the advantages of integrating bismuth into multifunctional nanoplatforms for real-time diagnostics.

Finally, Hernández-Rivera *et al.* (2017) advanced the concept of hybrid bismuth contrast agents through the functionalization of ultrashort carbon nanotubes (US-tubes) with Bi(iii) oxo-salicylate clusters.^91^ These Bi_4_C clusters were selected due to their superior X-ray attenuation relative to iodine and their favorable safety profile. The resultant nanohybrid exhibited efficient CT imaging capabilities, illustrating the feasibility of carbon-based delivery vehicles for heavy metal payloads in diagnostic imaging. This work adds another dimension to the design of BiNP-based CT contrast agents, particularly in harnessing high-aspect ratio nanostructures for improved circulation and targeting profiles.

The studies underline the rapid evolution and diversification of bismuth-integrated nanoparticle systems for advanced diagnostic imaging. From dual MRI/CT contrast platforms to multimodal theranostic constructs and molecular-scale agents, bismuth's high atomic number, tunable chemistry, and biocompatibility continue to drive innovations across biomedical imaging landscapes.

#### 3.3.2 Co-loading with immunotherapeutics or photothermal sensitizers

The strategic co-loading of bismuth-based nanoparticles (BiNPs) with immunotherapeutics or photothermal sensitizers has emerged as a powerful platform for enhancing cancer treatment efficacy, particularly in the context of synergizing photothermal therapy (PTT) with immunotherapy. This multifunctional approach is grounded in the unique properties of BiNPs, including their high X-ray attenuation coefficient, biocompatibility, and efficient photothermal conversion under near-infrared (NIR) irradiation.^92,93^ These features make BiNPs excellent candidates for both imaging and therapeutic applications. However, their therapeutic potential is significantly amplified when co-loaded with agents such as Toll-like receptor agonists and checkpoint inhibitors, or photothermal sensitizers like indocyanine green (ICG), which enable a cascade of immune responses initiated by localized tumor destruction. The idea behind this co-loading strategy is to convert the immunologically “cold” tumor microenvironment into a “hot” one, where tumor-associated antigens released from PTT-induced tumor cell death can be recognized and processed by antigen-presenting cells (APCs), such as dendritic cells, which are then further activated by immunoadjuvants encapsulated in the same nanoplatform. This dual-action mechanism fosters robust systemic anti-tumor immunity, inhibits metastasis, and even establishes long-lasting immunological memory capable of preventing tumor recurrence.^94,95^

**Fig. 11:**
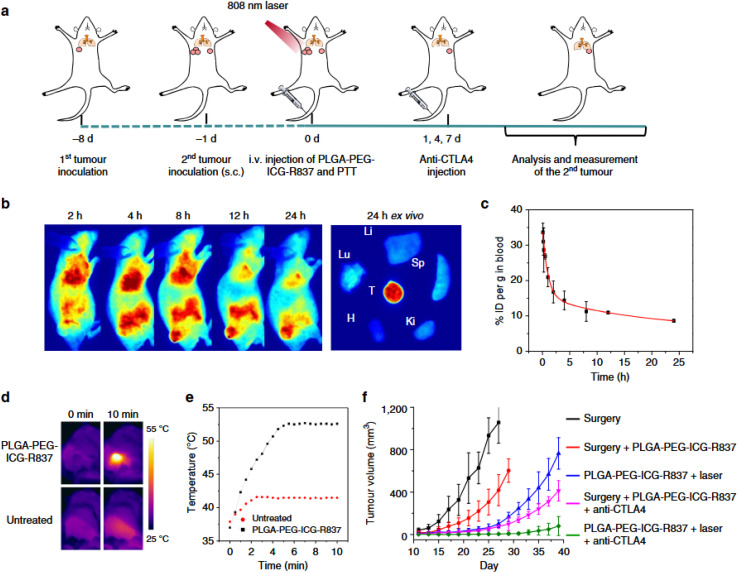
Photothermal therapy (PTT)-induced immunotherapy following systemic nanoparticle administration. (a) Schematic diagram of the experimental design in CT26 tumor-bearing mice. (b) *In vivo* fluorescence imaging of mice at various time points after intravenous (i.v.) injection of PLGA-PEG-ICG-R837, with corresponding ex vivo fluorescence images of major organs and tumors collected 24 hours post-injection. Abbreviations: Tu (tumor), Li (liver), Sp (spleen), Ki (kidney), H (heart), Lu (lung). (c) Blood circulation profile of PLGA-PEG-ICG-R837, determined by measuring ICG fluorescence in blood samples at designated time points post-injection (*n* = 3). (d) Infrared (IR) thermal images of CT26 tumor-bearing mice treated with PLGA-PEG-ICG-R837 or PBS under 808 nm laser irradiation (0.8 W cm^−2^). (e) Quantified tumor temperature changes derived from IR thermal imaging data in (d). (f) Growth curves of secondary tumors in different treatment groups of CT26 tumor-bearing mice following ablation of primary tumors (*n* = 6 per group). Data are shown as mean ± s.e.m.^92^

In this context, the work by Chen *et al.* (2016) exemplifies this synergistic strategy by demonstrating how co-encapsulation of ICG, a clinically approved photothermal agent, and imiquimod (R837), a Toll-like receptor 7 (TLR7) agonist, within a PLGA nanoparticle matrix can serve both therapeutic and immunological functions.^92^ While not based on bismuth, this model sets a strong precedent for BiNP-based co-loading platforms. Upon NIR laser irradiation, ICG mediates local hyperthermia, effectively ablating primary tumors and inducing immunogenic cell death that releases tumor-associated antigens. In addition to immunogenic cell death, markers of apoptosis such as cleaved caspase-3 expression and DNA fragmentation (typically detected *via* TUNEL assay) were also observed in treated tumor tissues, indicating that the combined photothermal-immunotherapy triggers both innate immune responses and programmed cell death mechanisms. Furthermore, the downregulation of anti-apoptotic proteins like Bcl-2 and the upregulation of pro-apoptotic factors such as Bax reinforce the involvement of intrinsic apoptotic signaling pathways in this therapeutic context. Concurrently, R837 promotes dendritic cell maturation and cytokine secretion, heightening immune surveillance. The subsequent administration of a CTLA-4 checkpoint blockade further augments T cell-mediated cytotoxicity, culminating in a highly potent systemic response capable of inhibiting metastasis and resisting tumor rechallenge. Importantly, the addition of polyethylene glycol (PEG) to PLGA in PLGA-PEG-ICG-R837 nanoparticles, as shown in Fig. 11, enhanced systemic delivery and tumor accumulation, allowing effective intravenous administration and sustained therapeutic outcomes without inducing systemic toxicity. The high accumulation in tumors, confirmed by fluorescence imaging (Fig. 11b), is critical for ensuring sufficient photothermal and immunogenic activity at the tumor site, and the elevated local temperature upon irradiation (Fig. 11d and e) effectively triggers both direct cytotoxicity and immune activation. Moreover, this approach significantly delayed secondary tumor growth compared to surgical removal and other control treatments (Fig. 11f), clearly illustrating the therapeutic advantages of this co-loading design.

The foundational concept of combining BiNPs with immunotherapeutic or photothermal agents benefits from the high atomic number of bismuths, which facilitates precise image-guided therapy through CT imaging, as well as their innate photothermal capacity. By integrating agents like R837 or ICG within the bismuth nanoparticle matrix or coating them onto the nanoparticle surface, researchers can create an intelligent delivery system that not only eliminates primary tumors through heat-induced ablation but also primes the immune system to recognize and destroy residual and metastatic tumor cells. This has been further validated by immune profiling in similar nanoplatforms, which shows elevated levels of effector memory T cells and pro-inflammatory cytokines like IFN-γ and TNF-α in treated mice, suggesting the generation of a long-lasting adaptive immune response. In parallel, apoptotic indicators such as annexin V staining and mitochondrial membrane depolarization in tumor cells have been reported in similar systems, reinforcing the dual contribution of immune-mediated and intrinsic apoptotic mechanisms in tumor suppression. The ability to engineer BiNPs that can be systemically administered and remain biocompatible, while achieving efficient co-delivery and controlled release of multiple therapeutic agents, is a major advancement in cancer nanomedicine. This finding underscores the critical role of co-loading strategies in maximizing the multifunctionality of BiNP-based platforms for cancer therapy. By uniting photothermal destruction with immune activation in a single, finely tunable nanosystem, this approach not only improves immediate therapeutic efficacy but also addresses the challenges of tumor metastasis and recurrence. The work of Chen *et al.* (2016),^92^ particularly their detailed evaluations illustrated in Fig. 11, offers a valuable framework for future designs of BiNP-based nanocomposites co-loaded with immunotherapeutic or photothermal agents, paving the way toward comprehensive, long-lasting, and minimally invasive oncological interventions.

In a study by Li *et al.* (2018), a theranostic platform was developed in which bismuth sulfide nanoparticles were encapsulated with mesoporous silica and loaded with the chemotherapeutic agent doxorubicin, followed by functionalization with trastuzumab to target HER-2 positive breast cancer.^85^ This sophisticated nanoplatform, Tam-Bi_2_S_3_@mPS, combined the deep tissue imaging capabilities of bismuth with precise tumor targeting and dual-modality therapy. The silica coating allowed for high drug-loading capacity, while trastuzumab enhanced tumor specificity. The core bismuth sulfide acted not only as a computed tomography (CT) contrast agent but also as a photothermal converter, enabling a synergistic effect between photothermal therapy (PTT) and chemotherapy. *In vivo* and *in vitro* analyses confirmed increased apoptosis in treated tumor cells, as evidenced by elevated levels of cleaved caspase-3 and enhanced TUNEL staining, indicating DNA fragmentation. These findings affirmed that the combined chemo-PTT strategy triggered intrinsic apoptotic pathways along with direct thermal damage.

Building upon this strategy, Zhao *et al.* (2018) introduced a biomimetic approach by cloaking hollow bismuth selenide nanoparticles with macrophage membranes and loading them with quercetin (a known heat shock protein inhibitor).^80^ The resulting M@BS-QE nanoparticles exhibited enhanced immune evasion and prolonged systemic circulation due to the macrophage-derived membrane, which facilitated tumor accumulation *via* CCL2-mediated recruitment and active homing. Upon near-infrared (NIR) irradiation, quercetin was selectively released, suppressing heat shock protein 70 (HSP70) to sensitize cancer cells to thermal ablation. Immunofluorescent staining revealed increased annexin V positivity and mitochondrial membrane depolarization, consistent with the initiation of apoptosis *via* mitochondrial pathways. Simultaneously, these nanoparticles downregulated signalling pathways associated with metastasis, such as *p*-Akt and MMP-9. The co-loading of a natural sensitizer and the use of immune cell mimicry highlighted a novel avenue for enhancing PTT efficacy and suppressing tumor spread.

In another immunotherapeutically inclined design, Guo *et al.* (2019) addressed the “don't eat me” signal employed by cancer cells by engineering PEGylated bismuth selenide nanoparticles conjugated with anti-CD47 antibodies (Ab-PEG-Bi_2_Se_3_).^67^ These particles effectively blocked the interaction between tumor-expressed CD47 and macrophage SIRPα, enhancing phagocytic activity. In addition to their potent NIR-induced photothermal effect, these nanoparticles enabled a dual-function platform where immune activation *via* checkpoint blockade was synchronized with thermal ablation. *In vivo* studies confirmed that Ab-PEG-Bi_2_Se_3_ led to efficient tumor eradication, with histological analyses showing increased infiltration of CD8^+^ cytotoxic T lymphocytes and upregulation of apoptotic markers such as Bax and cleaved PARP, pointing to immunologically mediated apoptotic cell death. This demonstrated the synergy between immunotherapy and PTT through immune checkpoint targeting.

The potential of augmenting PTT *via* autophagy inhibition was explored by Chen *et al.* (2019), who developed Bi@SiO_2_ nanoparticles loaded with chloroquine (CQ), an autophagy inhibitor.^93^ The silica layer not only protected the bismuth core from oxidation but also enhanced light scattering and photothermal performance. CQ delivery disrupted lysosomal degradation pathways in tumor cells, thereby inhibiting autophagy—a common resistance mechanism to PTT. Subsequent exposure to NIR irradiation resulted in significant apoptosis, as indicated by caspase activation and increased chromatin condensation observed *via* DAPI staining. This co-loading of a photothermal agent and autophagy modulator enabled tumor ablation under mild NIR irradiation, reducing off-target thermal damage while maintaining therapeutic efficacy. This study underscored the importance of intracellular regulatory pathways in shaping nanoparticle-assisted therapies.

The trend toward biocompatible and biodegradable platforms was reflected in the work by Liu *et al.* (2020), who synthesized bismuth nanoparticles using biologically derived proteins such as gelatin, bovine serum albumin, and human serum albumin 94. These BiNPs (BGPs, BBPs, and BHPs) offered multifunctionality, including CT and infrared thermal imaging capabilities, drug loading, and responsive drug release under acidic and NIR-triggered conditions. Treated tumor models demonstrated marked apoptotic activity, with flow cytometry revealing increased annexin V+/PI– cell populations and histological sections showing apoptotic bodies. This work bridged the gap between therapeutic efficacy and biosafety, presenting an environmentally friendly and pH-responsive vehicle for synergistic chemo-photothermal therapy (CPTT), reinforcing the necessity of biocompatibility in clinical translation.

Taking the co-loading strategy further, Bao *et al.* (2023) developed bismuth-core mesoporous silica nanoparticles (BMSNs) functionalized with a breast cancer-targeting peptide (AR) and loaded with doxorubicin (DOX).^95^ The AR-modified BMSNs exhibited superior tumor-homing capability, ensuring that the co-loaded DOX accumulated efficiently at the tumor site. These nanoparticles provided enhanced CT imaging and converted NIR light into localized heat to facilitate chemo-photothermal synergy. Apoptosis assays indicated significant activation of intrinsic apoptotic cascades, including the cleavage of caspase-9 and cytochrome c release from mitochondria, which, in conjunction with the cytotoxic effects of DOX, amplified tumor cell death. The dual-targeted approach not only improved tumor imaging but also amplified therapeutic outcomes through precise delivery and synchronized release of heat and cytotoxins.

Finally, Meng *et al.* (2023) addressed the limitations of weak drug loading and lack of multifunctionality in inorganic nanoparticles by constructing a gold-crowned bismuth-based nanocomposite (BACN).^96^ Although specific mechanistic details were truncated, the design likely integrated the superior optical and thermal properties of gold with the imaging and therapeutic advantages of bismuth. Similar hybrid nanocomposites have been associated with strong induction of apoptosis in cancer cells, often through oxidative stress-mediated mitochondrial damage and PARP cleavage. Such hierarchical co-loading platforms exemplify the next generation of multifunctional nanotherapeutics where photothermal sensitizers, imaging agents, and immunomodulators can coexist to orchestrate highly controlled and potent anti-cancer responses.

Together, these studies collectively affirm the transformative potential of co-loading bismuth-based nanoparticles with immunotherapeutics or photothermal sensitizers. Each system leverages unique targeting ligands, biomimetic coatings, or biological inhibitors to enhance tumor accumulation, modulate the immune system, and augment photothermal destruction. Crucially, the recurring observation of apoptotic markers—such as caspase activation, DNA fragmentation, and mitochondrial dysfunction—across these studies highlights the central role of programmed cell death in mediating therapeutic success. These integrated approaches create highly personalized, multi-modal therapeutic strategies capable of overcoming conventional treatment resistance.

## 4 Preclinical studies and advances

### 4.1 *In vivo* tumor inhibition

#### 4.1.1 BiNPs significantly enhance RT-CDT synergy, reducing tumor growth in mouse models

Bismuth-based nanoparticles (BiNPs) have emerged as promising agents for enhancing cancer treatment efficacy through the synergistic integration of radiotherapy (RT) and chemodynamic therapy (CDT). Owing to their high atomic number, excellent X-ray attenuation properties, and intrinsic catalytic activity, BiNPs provide a dual-function platform that not only amplifies radiation-induced damage but also initiates tumor-selective oxidative stress through Fenton-like reactions.^55^ When introduced into the tumor microenvironment, BiNPs catalyze the decomposition of endogenous hydrogen peroxide into highly reactive hydroxyl radicals (˙OH), a hallmark mechanism of CDT, which induces oxidative damage in cancer cells. Concurrently, the presence of BiNPs augments RT by increasing localized dose deposition, thereby intensifying DNA damage and impairing the repair mechanisms of tumor cells.^97^

*In vivo* studies using mouse models have demonstrated that the co-application of BiNPs with RT and CDT results in significantly greater tumor suppression compared to either treatment alone. The synergistic interaction between RT-induced ionizing radiation and BiNP-mediated ROS generation leads to amplified cellular apoptosis and inhibition of tumor proliferation.^98,99^ Notably, this therapeutic strategy is marked by minimal systemic toxicity and favorable biocompatibility, reinforcing the clinical translational potential of BiNPs in combinatorial cancer therapy. The integration of BiNPs into RT-CDT regimens enables a precise, localized therapeutic response that effectively overcomes the limitations of conventional monotherapies.^100^

**Fig. 12:**
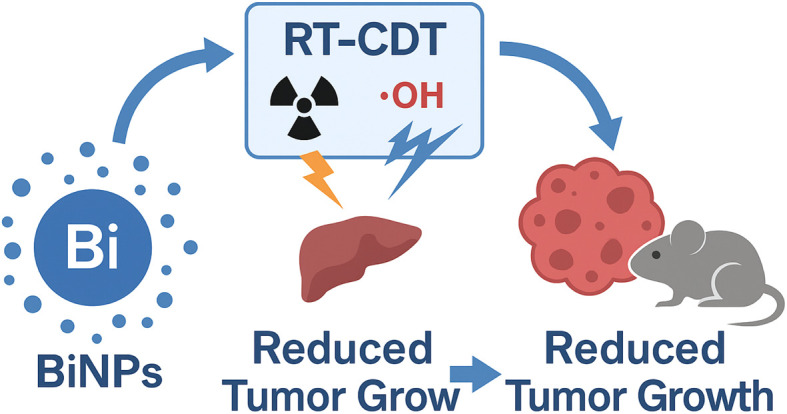
The synergistic effect of bismuth-based nanoparticles (BiNPs) in enhancing radiotherapy (RT) and chemodynamic therapy (CDT) to reduce tumor growth in mouse models.

The schematic representation of this mechanism is summarized in Fig. 12. The synergistic effect of bismuth-based nanoparticles (BiNPs) in enhancing radiotherapy (RT) and chemodynamic therapy (CDT) to reduce tumor growth in mouse models. The illustration depicts the targeted accumulation of BiNPs at the tumor site, their dual-functionality in promoting hydroxyl radical generation and radiation sensitization, and the consequential tumor volume reduction observed in preclinical studies. This integrated approach underscores the transformative potential of BiNPs in advancing next-generation oncologic nanomedicine.

The study by Cheng *et al.* (2020) presents compelling evidence that bismuth-based nanoparticles (BiNPs), specifically AgBiS_2_ nanoparticles, significantly enhance therapeutic synergy in the treatment of malignant tumors through a dual photothermal and photodynamic therapy (PTT/PDT) approach.^101^ While the study primarily centers on PTT/PDT, its findings also support the broader understanding that BiNPs are capable of enhancing multimodal therapies that rely on reactive oxygen species (ROS) generation and localized cytotoxicity—mechanisms that closely align with the principles underlying radiation therapy (RT) and chemodynamic therapy (CDT). In this regard, the AgBiS_2_-based nanoplatform represents an adaptable and potent nanoagent capable of amplifying therapeutic responses in aggressive tumor environments, akin to RT-CDT synergy.

**Fig. 13:**
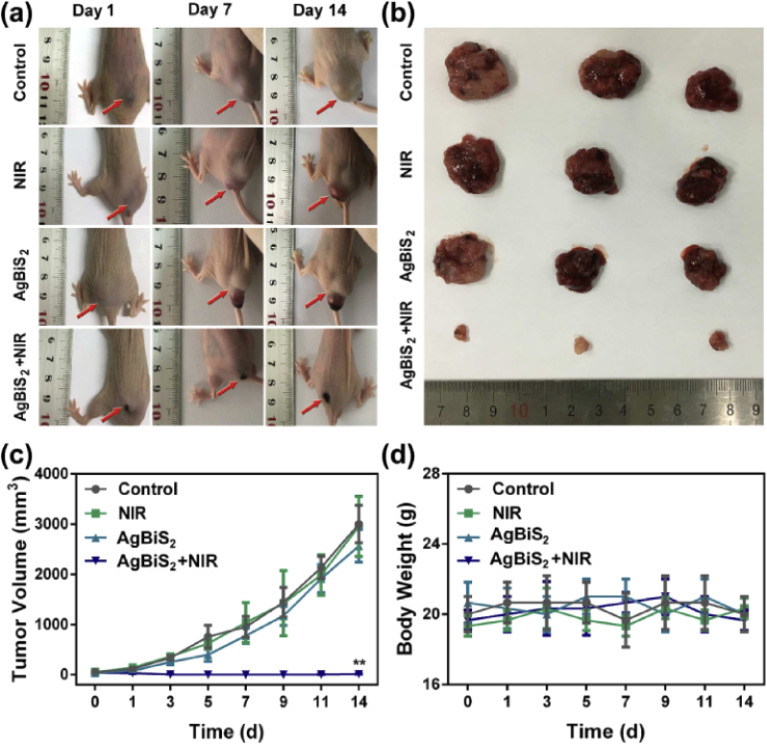
*In vivo* therapeutic outcomes across different treatment groups. (a) Representative photographs of osteosarcoma-bearing mice taken on days 1, 7, and 14 following various treatments, with tumor sites indicated by red arrows. (b) *Ex vivo* images of tumors excised from the mice 14 days post-treatment, showing visible differences in tumor size among the groups. (c) Tumor growth curves over time for each treatment group, illustrating significant differences in therapeutic efficacy. (d) Body weight changes recorded throughout the experimental period, reflecting systemic tolerance to the treatments. Data are presented as mean ± standard deviation (*n* = 3); *p < 0.05, **p < 0.01.^101^

The AgBiS_2_ NPs displayed an impressive photothermal conversion efficiency of 36.51% and significantly elevated intracellular ROS levels under near-infrared (NIR) irradiation, both of which are critical in facilitating cell apoptosis in tumor settings. In *in vivo* experiments using an osteosarcoma-bearing mouse model, the combination of AgBiS_2_ NPs and NIR laser irradiation completely suppressed tumor growth, while control groups receiving either component alone showed no significant tumor inhibition. As illustrated in Fig. 13, the AgBiS_2_ + NIR group exhibited full tumor growth suppression (Fig. 13b), whereas untreated and monotherapy groups experienced rapid tumor progression (Fig. 13a and c). Importantly, the PTT/PDT treatment had no observable adverse effects on mouse health, as indicated by stable body weight across all groups (Fig. 13d) and confirmed by histological analyses of vital organs.

Although not directly framed as RT-CDT, the ROS generation and thermal effects induced by AgBiS_2_ NPs under NIR irradiation share mechanistic similarities with CDT, where ROS-induced damage is central, and with RT, which also relies on radiation-activated oxidative stress and DNA damage. This overlap in biological response supports the assertion that BiNPs—by enhancing localized ROS production and thermal effects—can serve as synergistic agents in RT-CDT combinations. The metabolic and clearance profiles of these Bi-based NPs further reinforce their clinical promise; they were predominantly metabolized *via* the liver and spleen, with significant reductions in organ Bi content after seven days and caused no noticeable organ toxicity based on histological or biochemical analyses. The findings of Cheng *et al.* underscore the capacity of BiNPs to act as versatile therapeutic enhancers, with AgBiS_2_ nanoparticles demonstrating pronounced tumor inhibition through phototherapeutic mechanisms. These insights, particularly those depicted in Fig. 13, reinforce the growing consensus that BiNPs can be effectively leveraged to amplify multimodal oncologic therapies—including the synergistic action observed in RT-CDT protocols—while maintaining biocompatibility and minimizing systemic toxicity.

Guo *et al.* (2019) laid the foundation by demonstrating the multifunctional utility of CD47-targeted bismuth selenide nanoparticles (Ab-PEG-Bi_2_Se_3_) in augmenting photothermal therapy (PTT).^102^ While not a direct RT-CDT study, this work revealed the versatility of BiNPs in tumor immunomodulation through enhanced macrophage-mediated phagocytosis, which supports immune-mediated cytotoxicity synergistic with physical treatment modalities. This approach leverages the bismuth core for strong near-infrared (NIR) absorption and photothermal conversion, and the anti-CD47 modification effectively blocks the CD47–SIRPα axis, reducing immune evasion. These findings underscore how surface-functionalized BiNPs can integrate with other modalities, indirectly contributing to improved outcomes when used with RT or CDT.

Building on the radiosensitization capabilities of bismuth, Mishra *et al.* (2025) developed BiGd nanoparticles conjugated with *anti*-VEGF antibodies (aVEGF-BiGd) for targeted image-guided RT in hepatocellular carcinoma (HCC).^103^ These nanoconjugates displayed excellent biocompatibility and imaging capabilities while showing significant tumor reduction in RT-treated mouse models. The study reported enhanced apoptosis and suppressed tumor vascularity and proliferation, reflecting the combined radiotherapeutic and antiangiogenic effects. The inclusion of gadolinium provided additional imaging contrast, but the central role of bismuth in X-ray absorption and energy deposition made these nanoconjugates especially effective in amplifying RT responses—demonstrating that BiNPs can serve dual roles in diagnostics and treatment.

Liu *et al.* (2024) further innovated on this concept by introducing BiMn/BSA nanoparticles that exhibit multifaceted radiosensitization.^104^ Here, bismuth ensures high-*Z* radiation energy absorption, while manganese imparts catalase-like activity that decomposes endogenous H_2_O_2_ into oxygen, alleviating tumor hypoxia—a known resistance factor to RT. Notably, low-dose RT itself primed the TME by recruiting macrophages and inducing matrix metalloproteinase (MMP) activity, which in turn degraded the extracellular matrix, allowing deeper penetration of BiMn/BSA particles. This dynamic interplay resulted in complete tumor suppression, showcasing the strategic value of combining biological responses with rationally designed nanomaterials to optimize RT-CDT synergy.

In a complementary vein, Liu *et al.* (2021) devised a Cu^2+^-doped BiOCl nanoplatform (BCHN) capable of regulating the redox environment of tumors.^105^ The nanoparticles release H_2_O_2_ and consume GSH in the acidic TME, generating hydroxyl radicals *via* Cu-mediated Fenton-like reactions. These radicals amplify CDT-induced oxidative stress, while the liberated oxygen mitigates hypoxia, simultaneously enhancing RT responsiveness. Bismuth, as a high-*Z* element, augments radiation absorption, further compounding the antitumor effects. Thus, BCHN embodies an elegant integration of CDT and RT, where both modalities are mechanistically interdependent—hydroxyl radical generation and oxygenation sensitize tumors to radiation, while RT-induced DNA damage is made more lethal through oxidative insult.

Koo *et al.* (2022) advanced CDT strategies by developing CFp NPs—copper–iron peroxide-based nanoparticles—engineered to exploit TME acidity for controlled release and enhanced Fenton activity.^106^ Although devoid of bismuth, CFp NPs echo the conceptual themes seen in BiNP systems: localized radical generation, TME-responsiveness, and a feedback amplification loop where Cu^+^ catalyzes Fe^3+^ to Fe^2+^, promoting efficient hydroxyl radical production. Their therapeutic outcomes—complete tumor ablation at minimal doses—serve as a comparative benchmark and validate the clinical potential of similar BiNP-based systems, such as those by Liu and collaborators, which offer the added benefits of radiopacity and imaging-guided precision.

Feng *et al.* (2020) introduced ultrasound (US) as an auxiliary strategy to enhance CDT efficacy using bismuth ferrite oxide (BFO) nanocatalysts.^107^ These particles, activated by H_2_O_2_ and US stimuli, generate a robust ˙OH flux through turbulence-enhanced Fenton-like reactions.^108^ BFO nanoparticles demonstrated multimodal imaging capabilities and superior tumor suppression, reinforcing the importance of external energy triggers (like US or RT) in maximizing BiNP catalytic performance. Although not directly coupled with RT in this study, the mechanistic parallels with RT-induced ROS generation suggest a high compatibility for future RT-CDT combination protocols, where US could serve as an adjunct or substitute trigger in deep or inaccessible tumor regions.

Taken together, these interconnected studies reveal the emerging paradigm of bismuth-based nanoparticles as central agents in RT-CDT synergy. From immune engagement and antiangiogenic targeting to TME remodelling, hypoxia alleviation, and Fenton chemistry, BiNPs provide multifactorial therapeutic leverage. Each platform leverages the high atomic number of bismuths to potentiate radiation effects while simultaneously engineering the redox landscape of tumors to ensure sustained and selective cytotoxicity. These collective findings mark a pivotal advance in nanomedicine's capacity to integrate diagnostic, therapeutic, and immune-modulatory functions into single, responsive platforms that dramatically reshape the landscape of cancer treatment.

### 4.2 Imaging-guided therapy-BiNPs enable dual CT and photoacoustic imaging for treatment monitoring

Bismuth-based nanoparticles (BiNPs) are emerging as multifunctional agents in imaging-guided cancer therapy, offering dual-modality capabilities that significantly enhance treatment precision and monitoring. Due to the high atomic number (*Z* = 83) of bismuth, BiNPs possess excellent X-ray attenuation properties, making them highly effective as contrast agents for computed tomography (CT). This allows for high-resolution anatomical imaging with superior contrast compared to conventional iodine-based agents, enabling accurate tumor localization and real-time visualization during radiotherapy or drug delivery procedures.^29^ Furthermore, bismuth's strong photoacoustic signal generation, owing to its broad optical absorption in the near-infrared (NIR) region, provides complementary functional imaging through photoacoustic imaging (PAI), a technique that captures both structural and molecular information with high spatial resolution and depth.^67^

The integration of CT and PAI in a single BiNP-based platform supports multimodal imaging, enabling simultaneous anatomical and functional assessments. This dual capability allows clinicians to monitor biodistribution, tumor accumulation, and therapeutic response in real time, reducing uncertainty and allowing for timely therapeutic adjustments. For example, during combined radiotherapy-chemodynamic therapy, BiNPs not only facilitate radiosensitization but also permit real-time imaging of ROS generation and tumor hypoxia dynamics through PAI.^67^ Such comprehensive feedback enhances treatment planning, improves patient outcomes, and minimizes unnecessary tissue exposure. In addition to their imaging functionality, surface modification of BiNPs with tumor-targeting ligands and biocompatible coatings further enhances their accumulation in tumors *via* active or passive targeting mechanisms. These modifications increase imaging sensitivity while ensuring minimal systemic toxicity. As a result, BiNPs serve not only as effective diagnostic tools but also as real-time guides for therapeutic interventions, bridging the gap between imaging and therapy in precision oncology.^109^

**Fig. 14:**
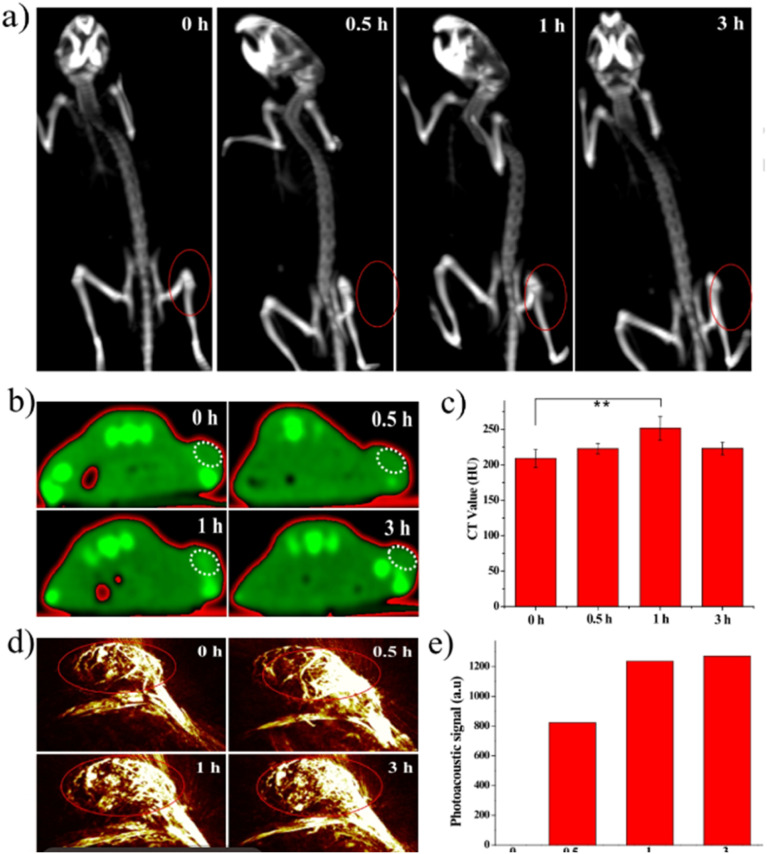
*In vivo* X-ray Computed Tomography (CT) and photoacoustic (PA) Imaging Performance of Gd-PEG-Bi nanoparticles (NPs). (a) Coronal and (b) axial CT images of tumor-bearing mice with pseudocolor enhancement, acquired before and after intravenous (i.v.) injection of Gd-PEG-Bi NPs. (c) Quantitative CT values of the tumor region pre- and post-injection (**p < 0.005). (d) *In vivo* PA imaging of the tumor at various time points post-injection, and (e) corresponding PA signal intensity quantification within the tumor area over time.^110^

For example, in the study by Wu *et al.* (2017), the development of Gd-PEG-Bi nanoparticles (NPs) presented a significant advancement in imaging-guided therapy by integrating dual-modality computed tomography (CT) and photoacoustic imaging (PAI) for effective treatment monitoring of cancer.^110^ This multifunctional design leverages the inherently high atomic number and X-ray attenuation coefficient of bismuth (Bi), enabling superior CT contrast compared to traditional agents such as iohexol and previously reported Bi_2_S_3_ nanostructures. As shown in Fig. 14a and quantified in Fig. 14c, Gd-PEG-Bi NPs significantly enhanced CT signals in glioma-bearing mice within one hour of intravenous injection. The tumor CT value rose from 210.2 Hounsfield Units (HU) pre-injection to 258.1 HU post-injection, demonstrating their strong tumor-specific accumulation and contrast enhancement. This enhancement was attributed to the enhanced permeability and retention (EPR) effect, which facilitated the passive targeting and prolonged retention of the nanoparticles within the tumor microenvironment.

In addition to CT imaging, Gd-PEG-Bi NPs showed remarkable performance in photoacoustic imaging. As illustrated in Fig. 14d and e, the PAI signal at the tumor site was negligible before injection but increased dramatically after administration of the NPs, reaching 822 arbitrary units (a.u.) at 0.5 hours and further intensifying to 1271 a.u. at 3 hours. This sustained signal amplification affirmed not only the efficient tumor localization of the NPs but also their high optical absorption in the near-infrared (NIR) range—critical for effective PAI contrast. The prolonged PAI signal provided additional evidence of the nanoparticles' retention within the tumor tissue due to the EPR effect, making them ideal for dynamic, real-time imaging over extended periods.

The integration of CT and PAI modalities in the Gd-PEG-Bi NPs offers a synergistic imaging platform that supports precise tumor localization, monitoring of therapeutic response, and early adaptation of treatment regimens. Unlike single imaging techniques that may provide limited spatial or functional information, this dual-modal approach enables both anatomical delineation (*via* CT) and high-resolution functional assessment (*via* PAI) within the same therapeutic timeline. As emphasized through the data in Fig. 14, such a strategy not only enhances the accuracy of cancer diagnosis and staging but also strengthens confidence in treatment outcomes by validating therapeutic efficacy in real time. This imaging-guided therapy paradigm is particularly valuable in tailoring individualized treatment plans, optimizing dosage, and ensuring timely interventions, thereby aligning with the goals of precision medicine.

In the study by Lu *et al.* (2018), the development of RGD-functionalized mesoporous silica-coated bismuth sulfide nanoparticles (RGD–Bi_2_S_3_@MSNs) introduced a highly promising theranostic platform that leverages dual-modality imaging—computed tomography (CT) and photoacoustic imaging (PAI)—to guide and monitor osteosarcoma (OS) therapy with remarkable precision.^111^ A key feature enabling this capability is the bismuth (Bi) core, which imparts a high X-ray attenuation coefficient critical for CT imaging, while also offering strong near-infrared (NIR) absorption conducive to photoacoustic signal generation.

**Fig. 15:**
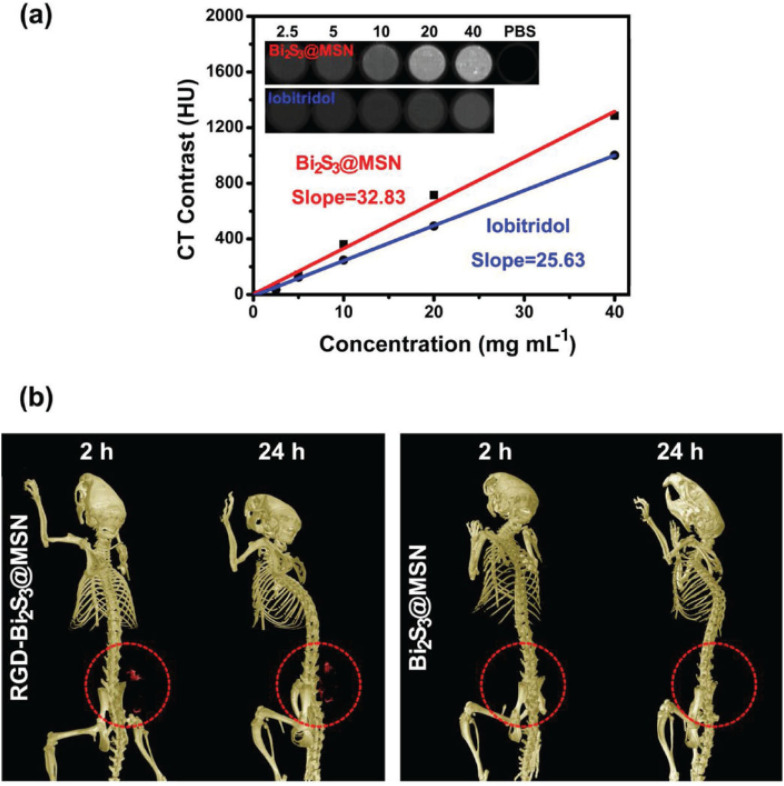
Computed Tomography (CT) Imaging Performance of the nanoparticles (NPs). (a) *In vitro* CT attenuation values (Hounsfield Units, HU) of Bi_2_S_3_@MSN compared with the clinical contrast agent iobitridol. *Inset:* Representative CT images of Bi_2_S_3_@MSN and iobitridol suspensions at varying concentrations. (b) *In vivo* CT imaging of UMR-106 tumor-bearing nude mice at 2 and 24 hours post intravenous injection of RGD–Bi_2_S_3_@MSN and Bi_2_S_3_@MSN (Bi_2_S_3_@MSN dosage: 5 mg kg^−1^). The tumor regions are indicated with red circles.^111^

The CT imaging performance of Bi_2_S_3_@MSNs was rigorously validated both *in vitro* and *in vivo*, as illustrated in Fig. 15. *In vitro* CT imaging (Fig. 15a) demonstrated a clear, linear increase in Hounsfield units (HU) with increasing nanoparticle concentration, reaching a slope of approximately 32.83 HU L g^−1^. This value significantly surpasses that of standard iodine-based clinical contrast agents such as iobitridol (25.63 HU L g^−1^) and iopromide (16.38 HU L g^−1^), underscoring the superior X-ray attenuation properties of Bi_2_S_3_. Such enhancement at lower dosages not only reduces the required contrast agent volume but also minimizes potential toxicity—an important consideration in clinical translation.

The *in vivo* CT imaging studies further established the nanoparticles' effectiveness for tumor-targeted contrast enhancement. As shown in Fig. 15b, the RGD-conjugated Bi_2_S_3_@MSNs, injected intravenously into osteosarcoma-bearing mice, resulted in a markedly stronger and more sustained CT signal at the tumor site compared to the non-targeted Bi_2_S_3_@MSNs. This contrast improvement was observable from 2 hours post-injection and continued to intensify up to 24 hours, a duration reflecting the prolonged blood circulation and effective tumor accumulation of the RGD-targeted nanocarriers. Notably, the control group (Bi_2_S_3_@MSNs without RGD) showed negligible CT signal at the tumor, emphasizing the crucial role of active targeting in achieving selective tumor imaging.Although not graphically detailed in the same figure, the dual-imaging nature of the Bi_2_S_3_@MSNs is also informed by their strong NIR absorption, which enables high-resolution photoacoustic imaging. This dual functionality allows for synergistic visualization of both tumor anatomy (*via* CT) and tumor physiology (*via* PAI), enabling a more holistic, real-time assessment of therapeutic progression. Together, these imaging capabilities support a closed-loop theranostic system—one that not only guides the delivery and distribution of therapeutics but also dynamically monitors therapeutic efficacy.

Through the results depicted in Fig. 15, the study convincingly demonstrates that RGD–Bi_2_S_3_@MSNs serve as a highly effective dual-imaging contrast agent. Their integration into a targeted, image-guided treatment paradigm for osteosarcoma represents a significant step toward precise, personalized oncology, wherein real-time imaging feedback directly informs therapeutic decision-making.

The collective body of work led by Zhou *et al.* (2021),^112^ Badrigilan *et al.* (2019),^113^ Yang *et al.* (2018),^114^ Zeng *et al.* (2020),^115^ Zhao *et al.* (2022),^116^ and Zhou *et al.* (2016),^117^ underscores the central role of bismuth-based nanoparticles (BiNPs) in enabling this dual imaging-guided therapy with high spatiotemporal precision and therapeutic efficacy.

Zhou *et al.* (2021) developed Bi-Ag@PVP nanoparticles, a silver-decorated bismuth heterostructure stabilized by polyvinylpyrrolidone (PVP), designed for simultaneous CT and PA imaging-guided phototherapy.^112^ The key innovation lies in the dual enhancement of both X-ray attenuation and NIR light absorption—facilitated by the high atomic number of bismuth (Z = 83) and the surface plasmonic properties of silver—which collectively amplify imaging sensitivity and treatment precision. CT imaging benefits directly from the increased X-ray attenuation of Bi, allowing for deep tissue visualization with excellent spatial resolution, while PA imaging is significantly strengthened due to enhanced photothermal conversion from the bimetallic architecture, yielding strong acoustic signals upon 808 nm laser irradiation. This synergistic capability enables real-time monitoring of both nanoparticle biodistribution and therapeutic progression, optimizing the therapeutic window for the subsequent photodynamic therapy (PDT) and photothermal therapy (PTT).

The design principles demonstrated by Zhou^112^ are conceptually echoed in Badrigilan *et al.* (2019),^113^ where a GQDs-Fe/Bi nanocomposite—comprising graphene quantum dots, superparamagnetic iron oxide (SPIO), and bismuth oxide (Bi_2_O_3_)—also facilitates dual-modal CT and MR imaging, although with PA functionalities inferred through NIR absorbance.^113^ While Badrigilan's work^113^ emphasizes CT and MR contrast, the high photo-to-thermal conversion efficiency (31.8%) and broad NIR absorbance imply potential for PA imaging enhancement, albeit not explicitly tested.^113^ The presence of Bi_2_O_3_ enhances CT signal strength, and SPIO provides T2-weighted MR contrast, showcasing the modular adaptability of bismuth in multimodal imaging frameworks.

Complementing this, Yang *et al.* (2018) refined the formulation by creating Bi@DLPC nanoparticles, wherein bismuth cores are surface-modified with the lipid DLPC for improved stability and biocompatibility.^114^ The dual imaging ability is again harnessed *via* the high X-ray attenuation for CT and robust NIR absorption for PA imaging. The reported photothermal conversion efficiency of 35% under 808 nm laser exposure not only enables efficient tumor ablation but concurrently generates strong PA signals, allowing deep-tissue imaging. Crucially, the enhanced permeability and retention (EPR) effect ensures tumor-specific accumulation, which was validated by distinct signal enhancements in both imaging modalities, directly guiding the therapeutic intervention.

Extending the conceptual versatility of BiNPs, Zeng *et al.* (2020) introduced a Bi_2_S_3_@Ce6–CeO_2_ nanocomposite, integrating a core of bismuth sulfide with photosensitizer chlorin e6 (Ce6) and cerium oxide for O_2_ evolution.^115^ Bi_2_S_3_ exhibits excellent CT and PA contrast owing to its intrinsic NIR absorption and high atomic number. The incorporation of Ce6 not only contributes to ROS generation for PDT but also amplifies the PA signal upon laser excitation, facilitating precise image-guided administration. The oxygen-evolving function of CeO_2_ addresses hypoxia-associated PDT resistance, thereby enhancing therapy efficacy, while the imaging functionalities remain robust for monitoring therapeutic response in real-time.

A significant advancement in probe miniaturization was presented by Zhao *et al.* (2022), who synthesized DNA-templated ultrasmall Bi_2_S_3_ nanoparticles (using ssDNA as a scaffold), tailored for PA imaging of myocardial infarction.^116^ Although the application is cardiac, the underlying mechanism—enhanced PA contrast due to bismuth's NIR photothermal responsiveness—reaffirms the central principle observed in oncological theranostics. The ultrasmall size facilitates deeper tissue penetration and prolonged circulation, enabling strong, localized PA signals with high sensitivity and negligible toxicity, highlighting the translatability of Bi_2_S_3_-based platforms for other deep-tissue imaging applications beyond cancer.

Reinforcing the multimodal imaging paradigm, Zhou *et al.* (2016) proposed FLBS-PFH-NPs, which combine folate-targeted perfluorohexane (PFH) with bismuth sulfide nanoparticles for dual ultrasound (US) and CT imaging.^117^ While PA imaging is not the focal point here, the CT imaging performance benefits from bismuth's high *Z*-value, and the acoustic contrast is enabled by the PFH core. The folate targeting and HIFU-triggered vaporization of PFH grant these nanoparticles image-guided, focused ultrasound therapy capabilities, foreshadowing further convergence of CT, US, and potentially PA in future hybridized imaging systems.

Collectively, these studies demonstrate a coherent evolution in the design of bismuth-based nanoparticles, converging around a central tenet: leveraging bismuth's intrinsic X-ray attenuation and NIR light absorption to enable simultaneous CT and PA imaging. Each successive design refines the balance between biocompatibility, imaging sensitivity, and therapeutic efficacy, offering comprehensive image-guided strategies for precise tumor localization, treatment planning, and post-therapy evaluation. The multifunctionality, tunability, and integrative imaging potential of BiNPs position them as premier candidates in the expanding landscape of image-guided nanomedicine.

### 4.3 Biocompatibility and safety profiles

Bismuth-based nanoparticles (BiNPs) have garnered significant attention in biomedical applications due to their unique physicochemical properties, including high atomic number, excellent X-ray attenuation, and favourable optical characteristics.^96^ These attributes make them suitable for various diagnostic and therapeutic modalities, such as computed tomography (CT), photoacoustic (PA) imaging, photothermal therapy (PTT), and photodynamic therapy (PDT). However, understanding their biocompatibility and safety profiles is crucial for clinical translation.^118^

#### 4.3.1 Biocompatibility and cytotoxicity

Studies on the cytotoxicity and biocompatibility of bismuth-based nanoparticles (BiNPs) have increasingly highlighted the dual nature of these nanostructures—where promising biomedical applications are tempered by potential toxicological concerns. Empirical evidence generally supports the notion that BiNPs exhibit low cytotoxicity across a range of normal and cancerous cell lines, especially at lower concentrations.^119,120^ However, toxicity becomes more apparent at elevated doses, with endothelial and epithelial cells demonstrating particular sensitivity. This underscores the need for precise dose optimization in therapeutic settings to balance efficacy with safety.^119^

Shakibaie *et al.* (2018) explored this balance by comparing the cytotoxicity of biogenic BiNPs with bismuth subnitrate against lung (A549) and breast (MCF-7) cancer cells, and normal fibroblast (3T3) cells.^120^ BiNPs displayed higher cytotoxicity in cancer cells (IC50 values of 10.9 and 35.4 μg mL^−1^, respectively) than in normal fibroblasts (IC50 of 42.8 μg mL^−1^), suggesting selective anticancer potential. However, bismuth subnitrate showed lower toxicity against cancer cells and higher toxicity in normal cells, indicating a narrower therapeutic window.

Further comparative studies, such as that by Liu *et al.*, revealed that BiNPs synthesized using bovine serum albumin induced minimal cytotoxicity in HUVEC and HepG2 cells, while slightly reducing viability in A549 and HEK293 cells at high concentrations (160 μg mL^−1^). Bismuth nitrate, by contrast, exhibited significantly greater toxicity under the same conditions, reaffirming the relatively safer profile of nanoparticulate forms over ionic counterparts.

Surface modifications have emerged as pivotal strategies to enhance biocompatibility. For example, coating BiNPs with polyethylene glycol (PEG), SiO_2_, or oligosaccharides has been shown to reduce protein adsorption and immune recognition, improving cellular tolerance. Luo *et al.* reported reduced cytotoxicity in HeLa and MG-63 cells when BiNPs were modified with SiO_2_ or PEG compared to unmodified or amine-functionalized counterparts. Wei *et al.* further demonstrated that oligosaccharide-coated BiNPs maintained cell viability above 80% even at 400 μg mL^−1^, illustrating the effectiveness of biocompatible surface engineering.

Mechanistic insights also suggest that BiNPs may trigger autophagy or oxidative stress-related pathways. Liu *et al.* observed autophagosome formation in HEK293 cells exposed to BiNPs and Bi^3+^ ions, characterized by increased LC3I/II expression. In a similar vein, Rodilla *et al.* found that exposure to bismuth-citrate led to hypoxia-like stress responses and upregulation of Bnip3, a pro-apoptotic protein, in alveolar macrophages. Gao *et al.* attributed BiOCl-induced cytotoxicity in HaCaT cells to G0/G1 cell cycle arrest and reactive oxygen species (ROS) generation, further supporting the role of oxidative stress in bismuth-related cytotoxicity.

Akbarzadeh *et al.* (2018) reinforced these findings through their investigation of bare and ligand-conjugated Bi_2_O_3_ NPs in KB and A549 cell lines.^121^ Their results showed that although both forms were internalized effectively, significant cytotoxicity was observed only above 50 μg mL^−1^, especially after 24-hour exposure. Apoptosis and ROS-mediated cell death were key mechanisms implicated in the cytotoxic response.

Finally, Akhtar *et al.* (2023) emphasized that Bi_2_O_3_ NPs generally exhibit measurable toxicity only beyond concentrations of 40–50 μg mL^−1^ across various epithelial cell lines.^119^ This threshold aligns with previous observations, underscoring a relatively consistent dose-dependent toxicity pattern.

Collectively, these studies reveal that while BiNPs offer selective anticancer effects and favorable cytocompatibility at controlled doses, their safety profile is highly dependent on concentration, surface chemistry, and cellular context. Strategic modifications, such as PEGylation and ligand conjugation, are instrumental in mitigating adverse effects, thereby enhancing the translational potential of BiNPs in oncology.

#### 4.3.2 Clinical translation and regulatory considerations

As of May 2025, bismuth-based nanoparticles (BiNPs) have not yet attained regulatory approval for clinical applications in cancer therapy, despite promising preclinical results that highlight their dual functionality as radiosensitizers and theranostic agents.^112–115^ A critical factor delaying clinical translation lies in the need to thoroughly understand and characterize the fundamental chemical and molecular mechanisms by which BiNPs enhance radiotherapy (RT) and chemodynamic therapy (CDT).^122,123^ Regulatory agencies require comprehensive mechanistic insights that explain not only efficacy but also safety, biocompatibility, and potential off-target effects, all of which must be supported by rigorous toxicological and pharmacokinetic data.^124^

The radiosensitization effect of BiNPs primarily stems from the high atomic number (*Z* = 83) of bismuth, which significantly increases the absorption of ionizing radiation such as X-rays. Upon irradiation, these nanoparticles emit secondary electrons that induce localized damage to tumor cell DNA, enhancing radiotherapeutic efficiency. This physical interaction initiates a cascade of molecular events wherein reactive oxygen species (ROS), particularly hydroxyl radicals (˙OH), are generated. These ROS inflict oxidative damage to critical biomolecules including nucleic acids, proteins, and lipids, ultimately triggering apoptotic and necrotic cell death pathways. Understanding the kinetics of ROS generation and cellular repair mechanisms is essential for anticipating not only therapeutic outcomes but also potential toxicities to surrounding healthy tissues, information that regulatory bodies rigorously scrutinize.

In parallel, chemodynamic therapy exploits the intrinsic catalytic properties of BiNPs to convert endogenous hydrogen peroxide (H_2_O_2_), often abundant in the acidic tumor microenvironment (TME), into highly reactive hydroxyl radicals through Fenton or Fenton-like reactions. This catalytic activity is frequently enhanced by incorporating transition metals such as copper (Cu^2+^) or manganese (Mn^2+^), which act as redox-active centers to accelerate ROS production. The resultant oxidative stress causes selective tumor cell death by disrupting redox homeostasis, depleting intracellular antioxidants like glutathione (GSH), and exacerbating DNA and protein damage. Moreover, the generation of oxygen from H_2_O_2_ decomposition alleviates tumor hypoxia, a major factor in radiotherapy resistance, thereby creating a favorable milieu for synergistic RT-CDT efficacy.^104,105^ Such interplay of biochemical pathways underscores the complex molecular choreography that BiNPs mediate within tumors.

Beyond ROS generation, BiNPs influence a range of molecular and cellular signaling cascades associated with apoptosis, autophagy, and immune modulation. For instance, exposure to bismuth compounds has been linked to upregulation of pro-apoptotic proteins such as Bnip3 and modulation of matrix metalloproteinases (MMPs), which remodel the extracellular matrix and facilitate deeper nanoparticle penetration into tumor tissues. These molecular events contribute to enhanced therapeutic responses but also raise important considerations regarding potential off-target effects and systemic toxicity, necessitating detailed investigations of biodistribution, metabolism, and clearance pathways. Such mechanistic understanding is crucial for regulatory evaluation, as it informs the safety margins and long-term biocompatibility profiles required for clinical approval.

The integration of imaging functionalities, such as computed tomography (CT) and photoacoustic imaging (PAI), further complicates the translational landscape. While these modalities offer invaluable real-time monitoring of nanoparticle distribution and therapeutic efficacy, their addition demands meticulous characterization to ensure that imaging agents do not interfere with the catalytic and radiosensitizing activities or introduce new toxicities. Regulatory agencies mandate that such multifunctional platforms undergo stringent assessment of each component's pharmacodynamics and pharmacokinetics, including immunogenicity and potential cumulative toxicity.^130^

Furthermore, dose optimization remains a pivotal challenge. Detailed knowledge of the chemical kinetics governing ROS production, H_2_O_2_ availability, and nanoparticle localization informs therapeutic windows that maximize tumor cytotoxicity while minimizing damage to healthy tissues. This balance is especially critical given the narrow therapeutic index of ROS-mediated therapies. Comprehensive mechanistic data thus facilitate rational dosing strategies that satisfy both clinical efficacy and safety requirements.

In light of these complexities, regulatory frameworks emphasize “safe-by-design” approaches that include surface modifications such as biodegradable coatings and tumor-targeting ligands. These modifications not only improve colloidal stability and biocompatibility but also enable controlled activation of catalytic functions specifically within the TME, thereby reducing systemic exposure and potential adverse effects.^124,125^ However, the interplay between surface chemistry, cellular uptake, and ROS generation must be carefully calibrated to preserve therapeutic potency while ensuring safety, a task that requires mechanistic insights at molecular and cellular levels. Ultimately, the successful clinical translation of BiNP-mediated RT-CDT depends on bridging the gap between intricate molecular mechanisms and practical therapeutic applications. Detailed mechanistic studies that elucidate how BiNPs interact with cellular redox systems, modulate immune responses, and influence tumor microenvironment dynamics are essential for constructing a comprehensive risk-benefit profile. These insights guide not only nanoparticle design and synthesis but also inform regulatory submissions, facilitating dialogue between researchers and authorities to accelerate the bench-to-bedside transition. As research progresses, incorporating visual summaries and mechanistic schematics within reviews and regulatory dossiers will further enhance understanding and acceptance of these innovative nanotherapeutics.

#### 4.3.3 Emerging clinical candidates of bismuth-based nanoparticles

Among the various classes of bismuth-based nanoparticles (BiNPs) under investigation, AGuIX-Bi emerges as the most advanced and clinically promising candidate currently moving toward translational application. AGuIX-Bi is a second-generation theranostic nanoparticle designed by integrating gadolinium and bismuth chelates onto a polysiloxane nanoparticle core. This combination strategically harnesses the high atomic number (*Z*) of bismuth to intensify local radiation dose deposition through the photoelectric effect, while leveraging gadolinium's imaging capabilities for real-time guidance. Recent work by Muradova *et al.* (2025) demonstrated that surface modification of AGuIX-Bi with cyclic Arg-Gly-Asp (cRGD) peptides, linked *via* a PEG spacer, significantly enhances tumor specificity.^131^ These peptides target RGD-binding integrins, which are highly overexpressed in numerous solid tumors, including non-small cell lung carcinoma (NSCLC).

*In vitro* results showed that AGuIX-Bi-cRGD nanoparticles exhibited enhanced internalization through integrin-mediated endocytosis and produced significantly greater radiosensitization than unmodified counterparts. *In vivo* evaluation using murine Lewis lung carcinoma models confirmed increased accumulation and tumor retention of the targeted nanoparticles, with no observable systemic toxicity. When combined with fractionated irradiation, AGuIX-Bi-cRGD nanoparticles facilitated a significant transformation of the tumor immune microenvironment. The so-called “cold” tumor milieu was converted into a “hot” immune-active state, characterized by the overexpression of HMGB1, a marker of immunogenic cell death, and a substantial increase in CD3+ CD8+ cytotoxic T-cell infiltration. This immunomodulatory effect led to delayed tumor progression and improved survival, supporting the potential of AGuIX-Bi-cRGD as a radiosensitizing-immunotherapeutic hybrid strategy for lung cancer.

Notably, the development of AGuIX-Bi capitalizes on the preclinical and clinical success of its predecessor, AGuIX®, which contains gadolinium alone and is already undergoing Phase I/II clinical trials in patients with brain metastases, glioblastoma, cervical cancer, and pancreatic or lung tumors.^132^ These trials validate the safety, biodistribution, and multimodal imaging potential of the AGuIX platform, thereby offering a regulatory and translational advantage for the bismuth-integrated version. Importantly, AGuIX-Bi is currently under evaluation in the nano-SMART clinical trial—a targeted effort focused on centrally located NSCLC, thus representing the first active clinical translation of a bismuth-containing nanoparticle system for cancer therapy.^132^

In addition to AGuIX-Bi, other BiNP platforms continue to show considerable preclinical promise and could become future candidates for clinical development. One such system is the ultrasmall silica-based bismuth-gadolinium nanoparticle developed by Detappe *et al.* (2017), which demonstrated robust MR/CT imaging enhancement and radiosensitization *in vivo* in NSCLC models. This theranostic agent showed effective tumor suppression and prolonged survival in murine models under clinical radiotherapy conditions, with minimal off-target toxicity.^133^ What distinguishes this system is its compatibility with current CT-guided radiation therapy protocols and emerging MR-guided techniques, thus making it adaptable to existing clinical workflows.

Beyond these early clinical-stage or translational candidates, a number of multifunctional BiNP constructs have been developed with sophisticated therapeutic capabilities that may position them for future human studies. The Bi@mSiO_2_@MnO_2_/DOX nanocomposite, reported by Zhao *et al.* (2021), integrates photothermal therapy (PTT), chemotherapy, and chemodynamic therapy (CDT) within a single nanoplatform.^51^ The mesoporous silica coating protects bismuth cores, while MnO_2_ catalyzes H_2_O_2_ decomposition in the tumor microenvironment to generate O_2_, reducing hypoxia and improving therapeutic outcomes. Concurrently, the heat generated by BiNPs under near-infrared (NIR) irradiation releases DOX, a chemotherapeutic agent, and facilitates CDT *via* hydroxyl radical (˙OH) production. This composite also provides multimodal imaging *via* CT and MR contrast, demonstrating its potential as a comprehensive theranostic agent.

Khosravi *et al.* (2024) explored the radiosensitizing efficacy of bismuth selenide (Bi_2_Se_3_) nanoparticles in colon cancer cells (HCT-116).^134^ Their *in vitro* findings revealed that Bi_2_Se_3_ NPs significantly increased radiation sensitivity in a dose-dependent manner, reducing cell viability and enhancing radiotherapy effectiveness. These results support Bi_2_Se_3_ NPs as a viable radiosensitizer candidate with potential for *in vivo* translation, pending further toxicological and pharmacokinetic studies. Xiao *et al.* (2024) introduced a Bi/Fe_3_O_4_@P3 nanocomplex, self-assembled using poly(aspartic acid), Bi^3+^, and ultrasmall Fe_3_O_4_ nanoparticles.^84^ This system provides dual-modal CT/MR imaging while enabling synergistic photothermal and chemodynamic therapy. The elevated intracellular H_2_O_2_ resulting from thermal stress amplifies the Fenton reaction, producing cytotoxic hydroxyl radicals that promote tumor cell death. In addition, Bi^3+^ depletes intracellular glutathione, further enhancing CDT efficacy. The integration of imaging and therapy in this construct showcases a rational design for minimally invasive, multimodal tumor eradication strategies, particularly for early-stage or microtumor lesions.

Collectively, these platforms represent a growing body of evidence supporting the emerging clinical potential of bismuth-based nanoparticles. While AGuIX-Bi currently stands as the most advanced candidate entering human trials, several others display clear multimodal, biocompatible, and tumor-specific capabilities that fulfill the prerequisites for clinical translation. Continued investment in mechanistic validation, toxicological profiling, and GMP-scale synthesis will be essential for converting these preclinical innovations into viable clinical therapeutics.

## 5 Challenges and strategic solutions

Radiotherapy-chemodynamic therapy (RT-CDT) based on bismuth-based nanoparticles (BiNPs) represents a compelling paradigm in multimodal cancer treatment, offering synergistic effects through the physical intensification of radiotherapy and the biochemical amplification of reactive oxygen species (ROS) within the tumor microenvironment. The high atomic number of bismuth (*Z* = 83) enables efficient absorption of ionizing radiation, thereby improving dose deposition in tumor tissues, while its catalytic properties facilitate Fenton-like reactions central to CDT. However, despite its promising therapeutic scope, a number of intrinsic barriers related to persistent cancer hallmarks—including tumor heterogeneity, hostile microenvironmental conditions, immune evasion, and therapy resistance—must be addressed to ensure successful clinical translation.

A primary obstacle to CDT efficacy is the heterogeneity of the tumor microenvironment (TME), particularly the variability in endogenous hydrogen peroxide (H_2_O_2_) levels. As CDT relies on the decomposition of H_2_O_2_ to generate toxic hydroxyl radicals *via* Fenton-like reactions, insufficient H_2_O_2_ in poorly vascularized or metabolically quiescent tumors can significantly limit ROS generation.^126^ To counter this, innovative strategies are emerging that involve the co-delivery of H_2_O_2_-producing enzymes or agents that reprogram tumor metabolism to elevate endogenous H_2_O_2_ levels. For instance, incorporating glucose oxidase (GOx) or targeting mitochondrial dysfunction could create a self-reinforcing redox cycle, enhancing the catalytic environment necessary for effective CDT. This biochemical conditioning of the TME not only enhances ROS production but also addresses therapy resistance associated with antioxidant-rich or hypoxic tumor regions.

Tumor heterogeneity, both at the inter- and intra-tumoral level, also presents a formidable challenge to uniform therapeutic responses. BiNPs offer a unique advantage in this regard due to their capacity for multifunctional customization. By engineering BiNPs with diverse surface ligands or tumor-specific antibodies, it becomes feasible to target multiple tumor subclones simultaneously. For instance, active targeting strategies—such as peptide, aptamer, or antibody conjugation—can address the heterogeneity of surface markers within a tumor mass.^95^ Furthermore, stimuli-responsive systems that activate selectively in response to acidic pH, redox imbalance, or enzyme expression unique to tumor tissues enhance the site-specific activation of BiNPs.^54^ This design adaptability allows BiNPs to bypass the limitations of one-size-fits-all therapeutics and instead offer precision engagement across diverse tumor phenotypes.

One of the most persistent challenges in oncology is immune evasion, whereby cancer cells escape immune surveillance through immunosuppressive signaling, altered antigen presentation, and the recruitment of regulatory immune cells. Recent findings suggest that BiNPs may help overcome this hurdle by promoting immunogenic cell death (ICD) and remodeling the TME. As discussed in the revised future prospects of this manuscript, BiNPs have demonstrated potential to activate dendritic cells, increase CD8+ T cell infiltration, and reverse immune suppression—hallmarks of “immune-hot” tumors. For example, GLP-conjugated BiNPs have been shown to induce dendritic cell maturation and cytokine release, contributing to improved T-cell priming and tumor antigen recognition.^131^ Similarly, transferrin-functionalized BiNPs used in glioma models not only enhanced radiation-induced DNA damage but also triggered ICD-related immune activation, illustrating their dual role as radiosensitizers and immunoadjuvants.^43^ By engaging the immune system, BiNP-based RT-CDT platforms may also reduce recurrence and metastasis, which are often fueled by immune escape mechanisms.

In terms of tumor resistance to therapy, particularly to conventional chemoradiation, BiNPs offer a tactical advantage through ROS-mediated cytotoxicity that is less reliant on specific molecular targets, thereby circumventing many mechanisms of drug resistance such as efflux pump expression or DNA repair pathway upregulation. Moreover, designing BiNPs with switchable surface coatings that respond to intracellular triggers can further enable timed and localized activation, reduce off-target effects and minimizing resistance development. For example, incorporating tumor-cleavable linkers or acid-sensitive bonds allows the outer shell to degrade specifically within the tumor milieu, unmasking the catalytic BiNP core at the site of action.^127^

However, addressing the biodistribution and systemic accumulation of BiNPs remains crucial. While bismuth's excellent radiopacity aids image-guided delivery, the heterogeneity of the enhanced permeability and retention (EPR) effect in human tumors often leads to variable nanoparticle accumulation.^95^ To enhance targeting efficiency, biomimetic strategies such as cell membrane cloaking or exosome-inspired delivery systems are being explored. These approaches can evade immune clearance and home in on tumors with greater specificity, enhancing therapeutic outcomes while minimizing systemic toxicity.

The potential long-term toxicity of BiNPs, particularly their retention in off-target organs such as the liver, spleen, or kidneys, must be rigorously assessed. Although studies suggest that BiNPs exhibit low acute toxicity at therapeutic doses, concerns remain about their fate post-treatment.^128,129^ To mitigate these risks, researchers are developing biodegradable or “self-destructive” BiNP designs, which degrade into non-toxic byproducts once their therapeutic role is fulfilled. Such constructs could leverage pH-sensitive disassembly or enzymatic cleavage to ensure efficient excretion after tumor engagement.

Real-time monitoring of BiNP behavior and therapeutic efficacy is another crucial frontier. Integration of diagnostic functionalities, such as CT contrast or photoacoustic signal enhancement, enables clinicians to track nanoparticle distribution and treatment response dynamically. This can improve decision-making related to treatment timing, dose adjustment, and early detection of therapeutic failure. However, incorporating these features introduces additional complexity to synthesis and regulatory approval, necessitating careful balance between functionality and manufacturability.^130^

Finally, the scalability and reproducibility of BiNP synthesis remain pressing challenges for clinical translation. Laboratory-scale synthesis often struggles with batch-to-batch variation, particle aggregation, or functional instability. Achieving uniform particle size, surface characteristics, and catalytic activity at scale will require development of good manufacturing practice (GMP)-compliant protocols and robust quality control systems. Furthermore, collaboration among nanomaterial scientists, oncologists, immunologists, and regulatory stakeholders is vital to harmonize BiNP design with clinical and safety standards. BiNP-based RT-CDT holds substantial promise in overcoming several persistent barriers that limit conventional cancer therapies. Through customizable targeting, TME responsiveness, immune activation, resistance circumvention, and theranostic integration, BiNPs offer a multifaceted solution to complex oncological challenges. If these technological and biological hurdles can be effectively addressed—through surface engineering, toxicity mitigation, real-time imaging, and scalable production—BiNP-enabled platforms may become cornerstones in next-generation precision oncology.

### 5.1 Future perspectives

The future of radiotherapy-chemodynamic cancer therapy (RT-CDT) utilizing bismuth-based nanoparticles (BiNPs) holds significant promise, particularly in the context of enhancing therapeutic efficacy while minimizing systemic toxicity. One of the most exciting prospects lies in the continued refinement of BiNPs as multifunctional platforms capable of simultaneous tumor imaging, radiosensitization, and reactive oxygen species (ROS) amplification. Advances in nanoparticle surface engineering are expected to play a critical role in enabling precise tumor targeting through ligand conjugation and stimuli-responsive release mechanisms. The incorporation of tumor-homing peptides, monoclonal antibodies, and responsive polymers could significantly increase the accumulation of BiNPs within the tumor microenvironment, thereby improving treatment selectivity and reducing off-target effects.

Moreover, the integration of bismuth's strong X-ray attenuation properties with advanced imaging modalities—such as computed tomography (CT) and photoacoustic imaging—is projected to support real-time monitoring of therapeutic outcomes. This image-guided capability could enable adaptive and personalized treatment regimens, where dynamic adjustments in radiation dose or chemodynamic activation are made in response to evolving tumor characteristics. The application of artificial intelligence (AI) and machine learning algorithms may further optimize this process by enabling predictive modeling of nanoparticle distribution, ROS kinetics, and patient-specific therapeutic windows.

From a materials perspective, ongoing research into the development of ultrasmall, biodegradable, or excretable BiNPs addresses current concerns related to long-term tissue retention and systemic toxicity. Environmentally sustainable approaches—such as green synthesis and the incorporation of biocompatible matrices like natural polymers or metal–organic frameworks (MOFs)—are anticipated to yield safer and more clinically viable nanostructures. These innovations could also accelerate regulatory acceptance by aligning with growing emphasis on biocompatibility and environmental safety in advanced medical materials.

Beyond their well-established role in radiosensitization and chemodynamic therapy (CDT), bismuth-based nanoparticles (BiNPs) are gaining recognition for their emerging potential to modulate the tumor immune microenvironment—a function that could profoundly enhance the scope and durability of radiotherapy (RT)-based interventions. Recent evidence suggests that BiNPs can initiate immunogenic cell death (ICD) and stimulate antigen presentation, which may convert immunologically “cold” tumors into “hot” ones, thereby enhancing their responsiveness to immunotherapies such as immune checkpoint inhibitors. This intersection of radiation physics, redox chemistry, and tumor immunology points toward a transformative paradigm in cancer therapy.

Huang *et al.* (2025) recently demonstrated the immunomodulatory effects of transferrin-functionalized bismuth nanoparticles (TBNPs), synthesized using a green chemistry route.^43^ Designed to overcome the challenges posed by the blood–brain barrier (BBB) in glioma treatment, these protein-coated TBNPs showed enhanced biosafety and improved tumor selectivity. Upon exposure to a clinically relevant 4 Gy dose of X-ray radiation, TBNPs induced substantial DNA damage in glioma cells. More notably, the study revealed that TBNPs triggered ICD, suggesting the potential to stimulate antitumor immunity *in situ*. The dual capability of these nanoparticles to both sensitize tumors to RT and promote immune activation resulted in effective tumor suppression in an orthotopic glioma mouse model through a synergistic radiotherapy–immunotherapy mechanism.

In a complementary study, Yu *et al.* (2019) explored the integration of BiNPs with a bioactive immunomodulator by conjugating bismuth sulfide nanoparticles with Ganoderma lucidum polysaccharide (GLP), resulting in GLP-BiNPs. These nanoparticles leveraged the high atomic number of bismuth to enhance X-ray absorption, while the GLP component significantly amplified immune responses.^131^*In vitro* assays demonstrated that BiNPs alone could mildly activate dendritic cells (DCs), but GLP-BiNPs markedly enhanced DC maturation, cytokine production, acid phosphatase activity, and T-cell proliferation. This dual action not only improved the efficacy of RT through increased radiosensitization but also initiated a cascade of immune events that contributed to tumor regression.

Yu *et al.* (2019) further reported that GLP-BiNPs improved tissue biodistribution by facilitating greater nanoparticle accumulation within tumor tissues and reducing off-target retention, particularly in the liver.^131^ Importantly, they observed a protective effect of GLP against kidney toxicity typically associated with bare BiNPs. Within 24 hours of administration, GLP-BiNPs significantly increased the population of mature DCs in both tumors and spleen, and subsequent analyses revealed heightened CD8+ T cell infiltration in the tumor microenvironment. The immune shift was further evidenced by an elevated serum IFN-γ/IL-4 ratio, indicating a more favorable Th1-type response. This was accompanied by effective suppression of primary tumor growth and a reduction in lung metastasis, highlighting the systemic reach of the immune activation.

Collectively, these findings emphasize the unique position of BiNPs at the interface of physical and biological therapy. Through radiosensitization, BiNPs intensify the local effects of radiation. *Via* CDT, they catalyze intracellular ROS generation, amplifying cytotoxicity within the acidic tumor milieu. Simultaneously, they can provoke immune activation through mechanisms such as ICD, DC maturation, and T-cell recruitment. These synergistic effects offer the prospect of not only localized tumor control but also systemic anticancer immunity, a hallmark of next-generation therapeutic strategies. Despite these promising outcomes, the molecular pathways underlying BiNP-induced immune modulation remain insufficiently understood. Further research is needed to delineate the roles of damage-associated molecular patterns (DAMPs), antigen-presenting cell activation, and adaptive immune engagement, particularly in the context of different tumor types and immunological landscapes. Large-animal studies and humanized models are essential to bridge the translational gap, enabling more accurate assessments of pharmacokinetics, biodistribution, and immune interactions.

As BiNPs increasingly embody multifunctional platforms—spanning diagnostics, therapeutic enhancement, and immune modulation—conventional regulatory categories may fall short. Their complex biological roles necessitate the development of adaptive regulatory frameworks that can address their hybrid character. Concurrently, interdisciplinary collaboration among materials scientists, immunologists, oncologists, and regulatory experts will be critical in refining nanoparticle formulations and optimizing delivery strategies. The convergence of nanotechnology, systems oncology, and immunotherapy underscores the future of BiNP-enabled RT-CDT as a central pillar of precision medicine. These platforms are poised to offer personalized, multimodal cancer treatment strategies that combine localized tumor ablation with durable immune surveillance. With sustained investment in translational research and supportive regulatory innovation, the clinical integration of BiNPs into the oncological landscape is not only plausible but increasingly inevitable.

## 6 Conclusion

Radiotherapy-chemodynamic therapy (RT-CDT) mediated by bismuth-based nanoparticles represents a promising paradigm shift in the multimodal treatment of cancer. By integrating the high X-ray attenuation and radiosensitizing capabilities of bismuth with its catalytic potential to amplify oxidative stress through Fenton-like reactions, this synergistic approach addresses several critical limitations of conventional monotherapies, including poor tumor selectivity, treatment resistance, and systemic toxicity. The unique physicochemical properties of various bismuth nanostructures—combined with advances in surface engineering, multifunctional integration, and imaging guidance—enable precise tumor targeting and real-time therapeutic monitoring. Preclinical investigations have consistently demonstrated enhanced therapeutic efficacy, improved tumor inhibition, and favorable safety profiles. However, for successful clinical translation, several challenges must be overcome, such as improving nanoparticle biocompatibility, ensuring reproducible large-scale synthesis, and addressing regulatory requirements. Continued interdisciplinary research focused on rational nanoparticle design, safe-by-design principles, and the incorporation of real-time imaging tools will be instrumental in accelerating the clinical adoption of BiNP-mediated RT-CDT. Ultimately, this innovative strategy holds the potential to transform cancer therapy by enabling more effective, targeted, and patient-tailored treatment outcomes.

## Conflicts of interest

The authors declare that they have no known competing financial interests or personal relationships that could have appeared to influence the work reported in this paper.

## Data Availability

This article is a review and does not contain any original experimental data. All data referenced and discussed in this manuscript are available from publicly accessible sources, including peer-reviewed publications and scientific databases, which are properly cited within the text. No new datasets were generated or analyzed during the current study.
